# Quantitative systems pharmacology model of B cell immune response in mouse

**DOI:** 10.3389/fimmu.2026.1745710

**Published:** 2026-04-30

**Authors:** Yaroslav Ugolkov, Alina Volkova, Gabriel Helmlinger, Kirill Peskov, Victor Sokolov

**Affiliations:** 1 Faculty of Bioengineering and Bioinformatics, Lomonosov Moscow State University, Moscow, Russia; 2 Marchuk Institute of Numerical Mathematics of the Russian Academy of Sciences (INM RAS), Moscow, Russia; 3 Modeling & Simulation Decisions FZ-LLC, Dubai, United Arab Emirates; 4 Quantitative Medicines, Lexington, MA, United States; 5 Research Center of Model-Informed Drug Development, I.M. Sechenov First Moscow State Medical University, Moscow, Russia

**Keywords:** antibody secreting cell (ASC), B cell immunity, MIDD (model-informed drug development), pharmacometric modeling, QSP model, quantitative systems pharmacology, systemic autoimmune diseases

## Abstract

B cell-mediated immunity plays a crucial role in long-term humoral protection. However, dysregulation of B cell development and differentiation may lead to the persistence of autoreactive clones, contributing to autoimmune diseases. Despite a number of therapeutic agents in preclinical and clinical development targeting B cell biology, several challenges still limit their successful translation into clinical use. To better understand B cell-targeting mechanisms of action quantitatively and mechanistically, we developed an integrative systems pharmacology model that describes T cell-dependent B cell response to antigen exposure in mouse. The model includes 20 ordinary differential equations representing key biological processes involved in the B lymphocyte response: B cell activation in secondary lymphoid organs; antibody-secreting cell (ASC) generation; migration to the bone marrow; and redistribution to peripheral tissues. The model adequately described ASC dynamics across tissues and IgG time profiles in plasma. Local and global sensitivity analyses identified the ASC production rate as the main contributor to cell counts in the spleen and in lymph nodes, while ASC levels in the bone marrow were primarily controlled by their influx rate, reflecting survival niche availability. Moderate variations of this influx rate parameter allowed the model to capture high inter-study variability in bone marrow ASC levels, explaining the observed heterogeneity in the functional immune response. The model can be further used as a quantitative tool to study B cell responses and their dysregulation in autoimmunity. It can be extended by integrating plasma-cell biology and autoantibody production, ultimately supporting the development of new therapeutic strategies for autoimmune diseases.

## Introduction

B cells represent a central component of the adaptive immune system, responsible for antibody production and immunological memory (1). Across the lifespan of an organism, B cell immunity undergoes a complex developmental process that ensures the generation of a diverse and self-tolerant repertoire, capable of recognizing a wide range of antigens (2). B cell development initiates in the bone marrow, where hematopoietic progenitors differentiate into immature B cells. Following positive selection, these immature cells differentiate into transitional type 1 (T1) B cells and exit the bone marrow to enter the peripheral circulation (3). Upon entering the spleen, T1 B cells further differentiate into transitional type 2 B cells and eventually into naive B cells (4). Under steady-state conditions, naive B cells continuously recirculate through secondary lymphoid organs (5). The ability of naive B cells to patrol multiple tissues is essential for pathogen surveillance and the maintenance of immune homeostasis. In the absence of antigenic stimulation, many of these cells undergo apoptosis in the spleen or lymph nodes, maintaining the balance between cell production and cell death (6).

Upon antigen recognition, naive B cells enter germinal centers in the spleen and lymph nodes, where they undergo clonal expansion and iterative rounds of proliferation before terminal differentiation into antibody-secreting cells (ASC) (7). These cells are the primary source of antibody production and a key element of protection against pathogens. ASCs subsequently migrate from the lymphoid tissues to specialized survival niches in the bone marrow, where they persist long term and continuously secrete antibodies (8). Additionally, ASCs can disseminate to non-lymphoid tissues such as the liver, lungs, and gastrointestinal tract (9).

ASC migration from secondary lymphoid organs to the bone marrow is a critical step in the establishment of long-lived humoral immunity. The bone marrow provides a unique microenvironment that supports plasma cell survival through interactions with stromal cells, cytokines, and access to limited survival niches (10). Consequently, effective trafficking and retention of ASC in the bone marrow are key determinants of sustained antibody production following infection or vaccination. Furthermore, a subset of naive B cells differentiates into memory B cells, enabling rapid and robust responses upon re-exposure to the same antigen (11). The coordinated processes of B cell development, activation, and differentiation are fundamental to both immediate and long-term immune reactions.

Beyond protective immunity, dysregulated B cell responses are central to the pathogenesis of allergic diseases. Allergic disorders are characterized by antigen-specific class switching, most notably toward IgE production. Aberrant B cell activation, enhanced plasma cell differentiation, and prolonged survival of antibody-secreting cells contribute to sustained allergen-specific antibody titers and chronic inflammatory responses in tissues such as the airways and the skin (12). In conditions such as allergic asthma, atopic dermatitis and food allergy, altered germinal center dynamics and memory B cell responses play critical roles in disease persistence and exacerbation (13, 14). In addition to intrinsic immune dysregulation, environmental factors and chemical exposures can profoundly modulate B cell function and humoral immunity (15). A growing body of evidence indicates that environmental contaminants may alter B cell activation, impair antibody responses, or induce immunosuppression (16). Such perturbations can reduce vaccine efficacy, modify germinal center dynamics, or disrupt long-lived plasma cell maintenance (17, 18). Also, B cells are increasingly recognized as key drivers of pathogenesis in various autoimmune diseases (19, 20). Inappropriate activation, survival, or differentiation of B cells can lead to the production of autoantibodies reactive to self-antigens and pro-inflammatory cytokine secretion, which may contribute to the breakdown of self-tolerance and tissue damage in autoimmune conditions. Systemic lupus erythematosus (21–23), rheumatoid arthritis (24, 25), systemic sclerosis (26) and other autoimmune disorders exhibit dysregulated B cell phenotypes, including expanded populations of ASC and altered memory B cells. Over the past decade, this has led to the development of therapies targeting multiple biological molecules involved in B cell immunity, e.g., CD20 (rituximab, ocrelizumab, ofatumumab), CD19 (inebilizumab), as well as cytokines such as BAFF and APRIL (belimumab, atacicept, ianalumab) that regulate activation, persistence, and survival of B cells. Recent innovative efforts include the use of bispecific antibodies that redirect T cells to eliminate B lineage cells (mosunetuzumab, teclistamab, blinatumomab), as well as genetically modified T cell therapies (ciltacabtagene autoleucel, axicabtagene ciloleucel) that induce broad depletion of B cells or plasma cells (27). Despite advancements in the development of such innovative therapeutic options, their limited efficacy and high risk of adverse events underscore the need for novel therapeutic approaches based on mechanistic and quantitative understanding of the role of B cells in autoimmune conditions (28, 29). In this context, mechanistic mathematical modeling offers a powerful approach to integrate experimental data from multiple sources, to gain insights into the complex dynamical behavior of B cell immunity. However, as highlighted in a recent review by Ugolkov et al. (30), there is a notable scarcity of models specifically focused on the T cell-dependent B cell response in autoimmune conditions. Among the 38 mathematical models analyzed across 13 autoimmune conditions, the vast majority were focused on T cell response, cytokine influence, or macrophage activity. This gap underscores the need for dedicated modeling efforts, to mechanistically elucidate the role of B cells in autoimmunity. The aim of the current study was to develop a mechanistic model of B cell response in mouse, in order to identify key determinants of ASC dynamics. This research is intended to serve as a foundation for future translational applications in autoimmune disease modeling and optimization of therapeutic strategies in clinical settings.

## Methods

### Systematic literature review and data collection

**Figure 1:**
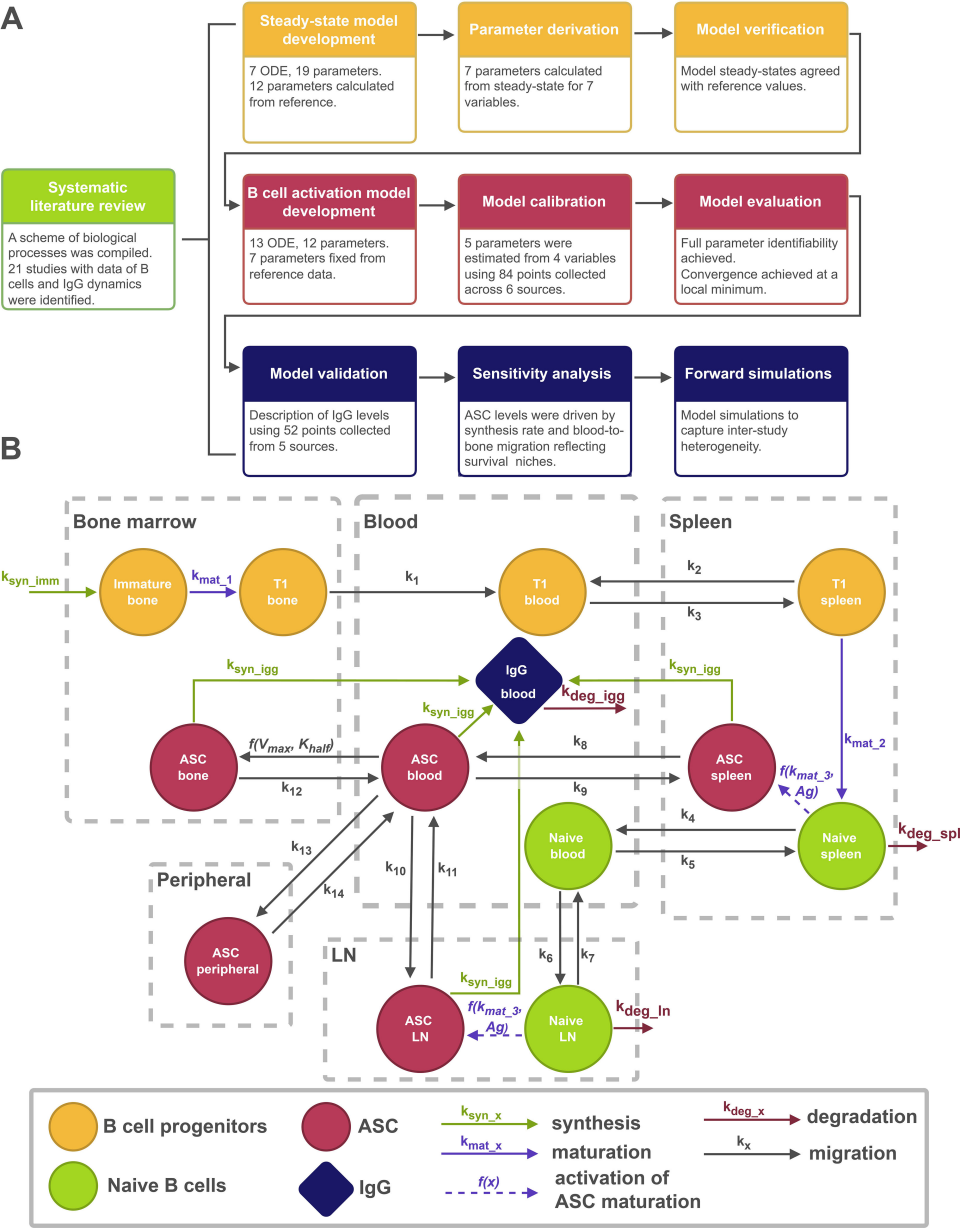
Model development workflow. **(A)** Workflow diagram. **(B)** QSP model scheme.

The overall study methodology combines a systematic review of the existing experimental data and a sequential model-based analysis represented in **Figure 1A**. This study does not include a separate systematic review of models, as this was already conducted in previous work (30). The most critical model variables were identified based on the current knowledge of immune system physiology available from published sources (1–3, 8, 25).

A systematic literature review was conducted in accordance with general guidelines (31). Quantitative data for model development were collected in two steps, whereby two authors (Y.U. and V.S.) independently screened all abstracts and summaries of publications containing relevant experimental data for eligibility.

Firstly, the literature search was conducted to collect quantitative data on non-activated B cell subpopulations under physiological conditions in mice. Studies were included if they reported absolute or relative cell counts in specific tissues, including bone marrow, spleen, lymph nodes, blood, and other relevant compartments, using flow cytometry or ELISA-based quantitative assays. Only data obtained from healthy, non-genetically modified animals were considered. No restrictions were applied regarding murine strain, age, or sex. Studies were excluded if they involved genetically modified or diseased animals, did not report quantitative cellular measurements, or lacked sufficient methodological description to allow data extraction. A special focus was put on the following cellular phenotypes: immature B cells, transitional B cells, and naive B cells, at steady-state. The final search query used in the PubMed database and Google Scholar on 25 August 2025 consisted of the following elements: (“immature B cell” OR “naive B cell” OR “transitional B cell”) AND (“bone marrow” OR spleen OR “lymph node” OR blood) AND (mouse OR murine).

In a second step, data describing ASC dynamics in T cell–dependent responses to antigen exposure were collected. Studies were included if they reported longitudinal measurements of ASC populations in blood, spleen, lymph nodes, or bone marrow following immunization in mice. The last update of literature searches were conducted using the PubMed and Google Scholar databases on 16 September 2025: (“antibody-secreting cell” OR “ASC”) AND (“bone marrow” OR spleen OR “lymph node” OR blood) AND (mouse OR murine) AND (activation OR differentiation OR migration OR homing OR survival OR immunization).

### Mathematical modeling

Model development was performed following existing good practices and workflows described for quantitative systems pharmacology (QSP) modeling (27–30). The model presented here consisted of 20 ODE and 31 parameters; it is represented in **Figure 1B**. All model equations and model code are provided in the Supplementary Materials (Supplementary Equations and Supplementary RxODE model code). Of these, 7 ODE and 19 parameters were used to describe a submodel of homeostasis, namely the B cell population at steady-state, in the absence of antigenic stimulation. Generation of immature B cells (*ImmBone, equation 8*) in the bone marrow, their maturation into T1 B cells (*T1Bone, equation 9*), migration to the spleen via the bloodstream (*T1Blood, equation 10*) and the generation of antibodies were described using 13 ODE and 12 parameters. In the modeled microenvironment of the spleen, T1 B cells (*T1Spleen, equation 11*) differentiated into naive B cells (*NaiveSpleen, equation 13*). Naive B cells continuously circulated across secondary lymphoid organs, including the spleen and lymph nodes (*NaiveLN, equation 14*), and were ultimately cleared in the spleen. All cell populations described in the homeostatic submodel were assumed to remain at steady-state levels. In response to antigen exposure, the activation component was represented via a time-dependent forcing function (*Antigen0*, *equation 15*). Antigen kinetics were described using a predefined parametric empirical function fitted to experimental antigen time-course data. Although expressed in differential form for numerical implementation within the ODE system, this formulation was adopted to ensure stable and reliable numerical integration of the coupled ODE system during simulations (**Supplementary Figure 1**). This function influenced the generation of ASC through six transit compartments (*Antigen1 - 5, Antigen, equation 16 - 21*). Upon antigen stimulation, naive B cells were activated and entered a proliferative and maturation phase within secondary lymphoid organs, prior to differentiating into ASC. In the present model, this activation–proliferation–differentiation cascade is represented in an aggregated effective ASC generation rate (*ASCSpleen, equation 22*, *ASCLN, equation 23*). Given the assumed low physiological precursor frequency of antigen-specific naive B cells (1 in 10^6) (32), activation does not explicitly deplete the total naive B cell pool, as the resulting reduction would be negligible and would not materially affect overall naive B cell dynamics in secondary lymphoid organs. The newly generated ASC migrated through the bloodstream (*ASCBlood, equation 25*) to the bone marrow (*ASCBone, equation 24*), where they may have persisted over a prolonged period of time. The parameter representing the sensitivity of ASC influx into the bone marrow to circulating ASC levels (*Khalf*) was set to 0.1, to reproduce the rapid accumulation kinetics observed experimentally (33–38). Although this small value mathematically implies near-maximal influx even at low circulating ASC numbers, the model maintained biological realism through the finite survival niche in the bone marrow. Together with the calibrated Vmax, this parameterization enabled the model to capture both fast migration and saturation-limited accumulation, consistent with observed ASC dynamics. The model also accounted for ASC redistribution into non-lymphoid organs, including the liver, the gastrointestinal tract, and the lungs (*ASCPeripheral, equation 26*); these organs were not directly involved in the adaptive immune response. ASC elimination was not included in the model: experimental data suggest their levels remain stable for up to 300 days post-immunization (36). In addition to these cellular dynamics, the model described the production of antigen-specific IgG antibodies (*IgGBlood, equation 28*). The total number of ASC in the bone marrow, blood, spleen and lymph nodes determined the IgG production rate (*equation 27*). ASC residing in peripheral non-lymphoid tissues are often tissue-adapted and support local antibody secretion within specific microenvironments; therefore, they were not included in the total ASC pool driving systemic IgG dynamics in the present model (39, 40).

Model calibration was performed using the maximum likelihood estimation method, in combination with the Nelder-Mead optimization algorithm (41). The objective function minimized was the -2 log-likelihood (-2LL), calculated as:


$$-2LL=\;\sum (\frac{residual^{2}}{\sigma^{2}}+\;\log(2*\pi *\;\sigma^{2}))\;$$

where the residual is the difference between observed and model-predicted values for each data point. The residual error ( σ) for each variable was calculated as $\sigma =\;\sqrt{\frac{\sum \;residual^{2}}{n}}$, where *n* is the number of observations for the corresponding variable. To ensure parameter positivity and to improve numerical stability during optimization, all calibrated parameters were log-transformed for fitting.

Practical identifiability of model parameters was assessed using relative standard error (RSE) values based on the Fisher information matrix (42) and likelihood profiling (43). To assess the uniqueness of a set of estimated parameter values, a multi-start fitting procedure was conducted using a distribution generated via Latin hypercube sampling, with parameter boundaries set at ±50% of the estimated parameter values. Model quality with respect to data description was assessed via various goodness-of-fit (GOF) plots, including observation versus prediction, weighted residuals (WRES) computed as $WRES=\frac{residual}{\sigma }$, WRES versus independent variable, WRES versus dependent variable, and WRES distribution (44). To assess uncertainty in model simulations, parameter sets were sampled from a multivariate normal distribution, defined by the estimated parameter values and the associated variance-covariance matrix, itself obtained during model calibration using the inverse Hessian matrix, computed using the optim function in R Statistics. A total of 1000 simulations were performed; resulting model trajectories were used to generate 95% confidence intervals for the predicted outputs. The 95% confidence intervals represent uncertainty in parameter estimates and do not account for inter-individual biological variability or residual experimental noise. Therefore, the simulated CI should not be interpreted as a prediction interval for individual experimental observations.

### Software

Data digitization was performed using WebPlotDigitizer (45). The workflow diagram and model scheme were created using the online tool draw.io (46). Data visualization was carried out using the R packages *ggplot2* (version 3.5.1), *cowplot* (version 1.1.1), and *gridExtra* (version 2.3). Model development and simulations were performed using *nlmixr2* (version 2.0.9); global sensitivity analyses were conducted using *sensitivity* (version 1.30.1), and model optimization was performed using the *optim* function from the *stats* (version 4.2.2) package in R Statistics (version 4.2.2).

## Results

A stepwise modeling strategy was applied to progressively build a quantitative framework of B cell dynamics. First, a steady-state model of naive B cell homeostasis was developed, followed by parameter derivation and model verification. Next, a dynamic model of B cell activation in response to antigen exposure was built, calibrated and evaluated. The modeling workflow further included validation against independent IgG kinetics data, sensitivity analyses, and model-based simulations to explore inter-study heterogeneity (**Figure 1A**). All 21 studies used for model development, parameterization, calibration, and validation are summarized in **Supplementary Table 1**. In the following sections, we present the results obtained at each stage of this workflow.

### Model calibration

Out of 31 parameters, 19 parameters were fixed based on experimental data, 7 parameters were derived from steady-state cell counts, and the remaining 5 parameters were estimated based on the available experimental data. The number of naive B cells activated upon antigen encounter were calculated as naive B cell/10^6, reflecting an estimated physiological precursor frequency of approximately 1 in 10^6^ antigen-specific naive B cells, as reported by Abbott et al. (32). Antigen dynamics were incorporated into the model using a function describing generalized antigen kinetics (33, 47). Experimental data demonstrated a consistent temporal shift between the maximum antigen exposure (viral load) (**Supplementary Figure 1**) and the maximum ASC response. To reproduce this distributed delay, six transit compartments were introduced. A mean transit time of 18 days was selected to match the observed average time to reach the maximum ASC counts (Tmax) (17.4 ± 9.45 days) (33, 41–43, 48, 49). This structure provides a flexible representation of delayed immune activation rather than imposing a fixed discrete lag. The half-saturation constant (*Khalf*) for ASC influx into the bone marrow was fixed at 0.1 days, since rapid saturation of ASC in the bone marrow was observed in the data used for calibration (33–38). The efflux rate constant from peripheral organs (*k14*) was assumed to be equal to the efflux rate constant from the bone marrow (*k12*), since ASC can persist over time in these compartments (50, 51). To calculate the total ASC count in the bone marrow, the number of cells from two femur bones was taken, multiplied by a coefficient of 7.9, to yield a femur-derived marrow of 12.7% vs. total bone marrow in mice, consistent with Katz et al. (52). The ASC count in all lymph nodes was estimated by multiplying the cell count from a single lymph node by 22, the total number of lymph nodes in mice (53). Seven parameters (*k1, k3, k5, k7, kmat_1, kmat_2, kdeg_spl*) were derived analytically using steady-state calculations. For each relevant equation, the time derivative was set to zero, and the resulting algebraic expression was solved for the unknown parameter using experimentally observed steady-state cell counts for this equation.

All other model parameters were calibrated based on the experimentally observed dynamics of ASC in response to antigen exposure (*k9, k12, k13, kmat_3, Vmax*). The RSE for all estimated parameters did not exceed 24%, indicating acceptable identifiability and precision. A summary of model parameters values is shown in **Table 1**.

**Table 1:** Final model parameter estimates.

Parameter	Value	RSE, %	Units	Description	Reference
Parameters for non-activated B cell steady-state					
				Algebraic steady-state parameters	
*ImmBone_ss*	1.97 * 10^6	–	cell	immature B cells in bone	calculated from (54)
*T1Bone_ss*	0.76 * 10^6	–	cell	T1 B cell in bone	calculated from (54)
*T1Blood_ss*	0.004 * 10^6	–	cell	T1 B cell in the blood	calculated from (55)
*T1Spleen_ss*	4.40 * 10^6	–	cell	T1 B cell in the spleen	calculated from (56)
*NaiveSpleen_ss*	24.30 * 10^6	–	cell	Naive B cell in the spleen	calculated from (4, 56)
*NaiveBlood_ss*	4.64 * 10^6	–	cell	Naive B cell in the blood	calculated from (57)
*NaiveLN_ss*	1.90 * 10^6	–	cell	Naive B cell in LN	calculated from (58)
				Rate constant of migration	
*k1*	26.32	–	1/day	T1 B cell from bone to blood	calculated from steady-state
*k2*	1.01		1/day	T1 B cell from spleen to blood	calculated from (59)
*k3*	6111	–	1/day	T1 B cell from blood to spleen	calculated from steady-state
*k4*	10.08	–	1/day	Naive B cell from spleen to blood	calculated from (60)
*k5*	73.53	–	1/day	Naive B cell from blood to spleen	calculated from steady-state
*k6*	4.9	–	1/day	Naive B cell from blood to LN	calculated from (60)
*k7*	62.62	–	1/day	Naive B cell from LN to blood	calculated from steady-state
				Other rate constants	
*ksyn_imm*	20 * 10^6	–	cell/day	Synthesis rate constant of immature B cell in bone	calculated from (61)
*kmat_1*	10.15	–	1/day	Rate constant of maturation immature B cells to T1 B cell	calculated from steady-state
*kmat_2*	4.55	–	1/day	Rate constant of maturation T1 B cell to naive B cell	calculated from steady-state
*kdeg_spl*	4.78	–	1/day	Elimination rate constant of naive B cell from spleen	calculated from steady-state
*kdeg_ln*	0.05	–	1/day	Elimination rate constant of naive B cell from LN	calculated from (62)
Parameters for activated B cell					
				Rate constant of ASC migration	
*k8*	2.52	–	1/day	from spleen to blood	calculated from (63)
*k9*	8.53	13.14	1/day	from blood to spleen	estimated from (33–38)
*k10*	143.60	–	1/day	from blood to LN	assumed on (64)
*k11*	2.72	–	1/day	from LN to blood	calculated from (63)
*k12*	0.02	23.23	1/day	from bone to blood	estimated from (33–38)
*k13*	16.26	23.55	1/day	from blood to peripheral	estimated from (33–38)
*k14*	0.02	–	1/day	from peripheral to blood	assumed on (50, 51)
				Other rate constants	
*ksin_igg*	22.00	–	pg/cell/day	Synthesis rate constant of IgG by ASC	calculated from (65)
*kdeg_igg*	0.12	–	1/day	Elimination rate constant of IgG	calculated from (66)
*kmat_3*	294.19	11.39	1/day	Rate constant of maturation naive B cell to ASC	estimated from (33–38)
				Other parameters	
*Vmax*	490.60	19.51	cell/day	The size of the survival niche in the bone marrow	estimated from (33–38)
*Khalf*	0.10	–	cell	Sensitivity of throughput in the bone marrow to accumulation ASC in blood	calculated from the observed data (33–38)

ASC, antibody-secreting cells; LN, lymph nodes; IgG, Immunoglobulin G; T1 B cell, transitional type 1 B cells; RSE, relative standard error.

**Figure 2:**
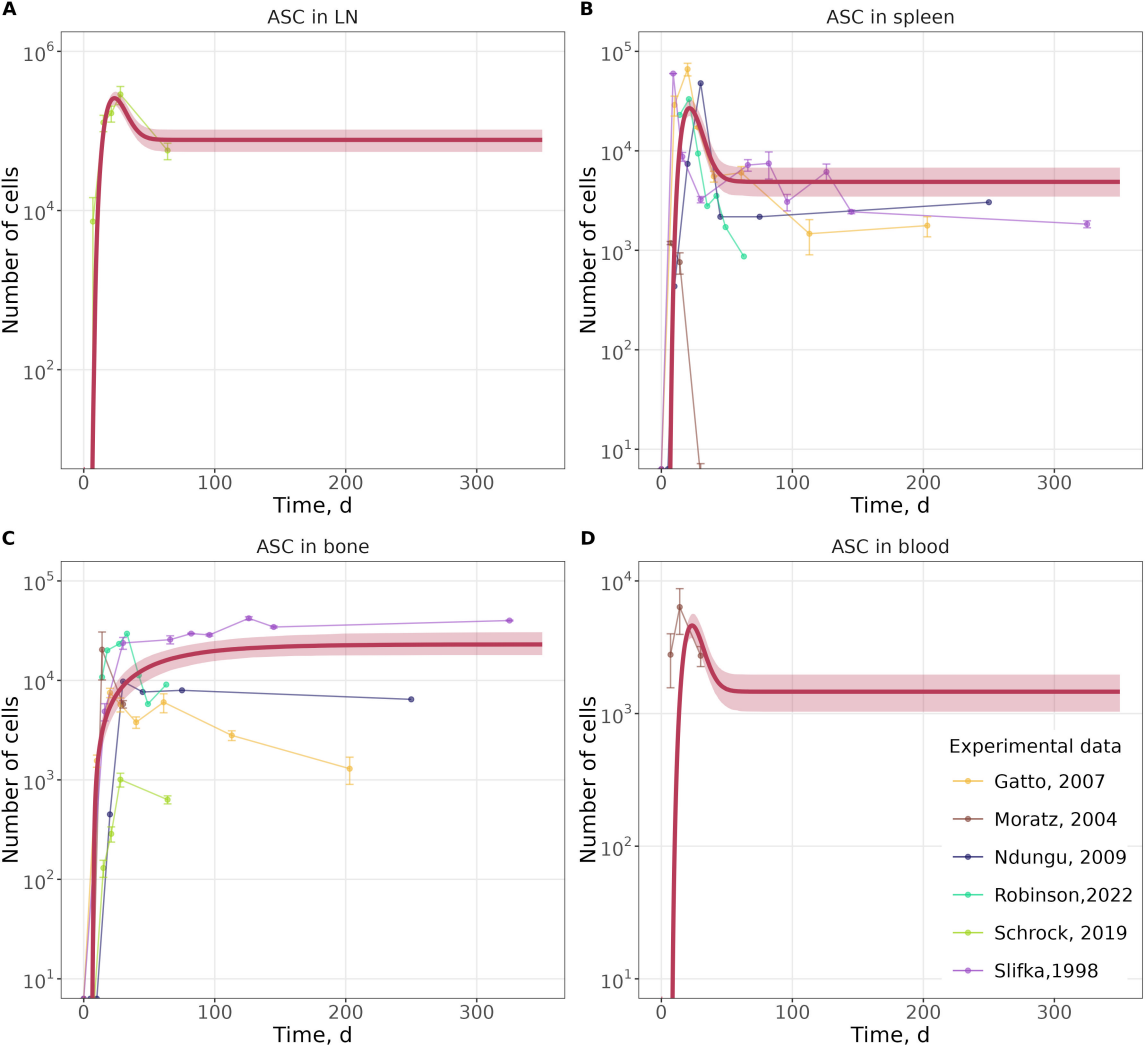
Model calibration against data, describing ASC dynamics in lymph nodes **(A)**, the spleen **(B)**, the bone marrow **(C)**, and blood **(D)**. Red lines - model predictions with 95% confidence intervals; color lines with points - experimental data. Error bars reflect the variability measure reported in the original publications.

As shown in **Figures 2A–D**, the model successfully reproduced experimental data for ASC counts across multiple tissues including spleen, lymph nodes, bone marrow, and blood. In the lymph nodes, ASC numbers were predicted to reach a maximum cell count (Cmax) of 2.6 * 10^5 cells (95% CI: 2.1 * 10^5–3.1 * 10^5), compared to an observed average of 2.9 * 10^5 (SE ± 4.3 * 10^4) cells (**Figure 2A**). In the spleen, the model predicted a Cmax of 2.7 * 10^4 cells (95% CI: 2.2 * 10^4–3.2 * 10^4), whereas experimental data indicated an average Cmax of 4.2 * 10^4 (SE ± 2.6 * 10^4) cells (**Figure 2B**). In the bone marrow, ASC levels gradually accumulated to a steady-state around 2.3 * 10^4 cells (95% CI: 1.8 * 10^4–3.0 * 10^4), consistent with experimental late timepoint counts of 1.6 * 10^4 (SE ± 2.1 * 10^4) cells (**Figure 2C**). In the blood, the model estimated a Cmax of 4.6 * 10^3 cells (95% CI: 3.7 * 10^3–5.6 * 10^3), while experimental data reported 6.4 * 10^3 cells (SE ± 1.4 * 10^3) (**Figure 2D**).The model predicted Tmax in lymph nodes at Day 24, close to the experimentally observed Day 28 (**Figure 2A**). In the spleen, the model Tmax occurred at Day 21, while experimentally it was observed at Day 17 (SE ± 9.45) (**Figure 2B**). In blood, the model Tmax was at Day 24, versus Day 14 in experimental measurements (**Figure 2D**). In the bone marrow, ASC accumulation was slower, reaching steady-state at Day 200 (**Figure 2C**). The differences in Tmax timing likely reflect variability in experimental sampling schedules as well as biological heterogeneity across studies.

Overall, the heterogeneity observed in experimental data is likely due to biological variability and inter-study differences, which are not explicitly represented in the current modeling framework; despite such limitation, trends in both Cmax and Tmax across tissues were adequately captured by the model.

Goodness-of-fit diagnostics (**Supplementary Figure 2**) further confirmed the ability of the model to describe the observed data. In the residual plots (**Supplementary Figure 2A**), 94.5% of weighted residuals (WRES) were found within the range of -2 to 2 over time, which corresponds approximately to the 95% confidence interval under the assumption of normally distributed residuals with mean of 0 and variance of 1. In the WRES vs. observed value plot (**Supplementary Figure 2B**), only a few residuals fell below -2. The observed vs. predicted plot (**Supplementary Figure 2C**) demonstrated a strong agreement between model predictions and experimental data. Moreover, the histogram of WRES (**Supplementary Figure 2D**) for all model variables approximated a normal distribution, indicating a well-calibrated residual structure.

To evaluate the stability of the estimated parameter values, a multi-start fitting procedure was applied. Upon varying the initial estimates of parameters within a ±50% range, the resulting optimized values deviated by no more than 15%, and the corresponding -2 log-likelihood values remained virtually unchanged, indicating robustness of the estimated parameter set (**Supplementary Figure 3**). Likelihood profiling was performed, in order to further explore parameter identifiability and ensure convergence of the calibration procedure. For all estimated parameters, the profiling curves confirmed that the selected values corresponded to the minimum of the log-likelihood function (**Supplementary Figure 4**). Confidence intervals were derived from the profiling results, with boundaries defined by an increase in -2 log-likelihood (which corresponds to the 95% confidence threshold). In all cases, these confidence intervals were relatively narrow, with upper and lower bounds deviating by no more than fivefold from the optimal parameter.

### Model validation

Model validation was performed using independent dynamic data on IgG plasma levels following immunization in mice, collected from 5 experimental studies (34–36, 67, 68). In order to standardize IgG levels reported in different studies, all values were normalized to the maximum concentration observed in each study (69, 70). Model performance was assessed by calculating the root mean square error (RMSE) between model predictions and experimental observations, and an analysis of weighted residual distribution over time.

**Figure 3:**
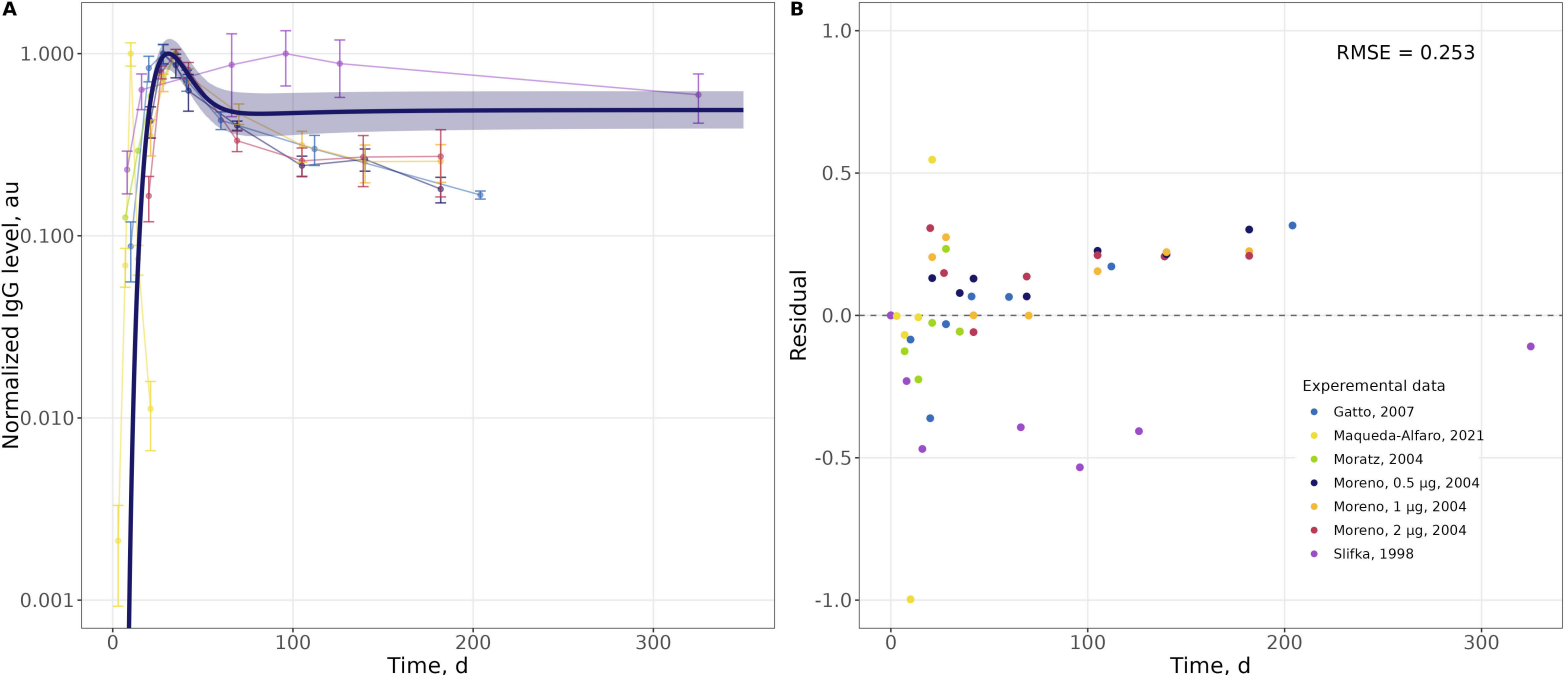
External model validation with independent data on IgG levels. **(A)** Line graph of normalized IgG levels over time; **(B)** scatter plot of residual values versus time. The blue line represents the model prediction with 95% confidence intervals; colored lines with points represent experimental data. Error bars reflect the variability measure reported in the original publications.

As shown in **Figure 3**, the model adequately described the time course of IgG concentration: a maximal reach in concentration, and a subsequent decline. More specifically, the model estimated that IgG levels rose to a maximal level at Day 30, while the experimental data showed a kinetically similar behavior with a maximum value at Day 38. The overall deviation between model predictions and observed values was quantified using the RMSE, which was equal to 0.253. However, it should be noted that some of the experimental IgG data showed a gradual decrease in IgG levels over time. Interestingly, when comparing this trend with the ASC dynamics from the same studies (**Figure 2C**), ASC levels remained relatively stable after reaching a plateau. Since the model assumed a constant secretion rate per ASC and fixed IgG clearance, it did not fully reproduce this late-phase decrease. This discrepancy indicates that the rate of antibody secretion per ASC may be variable across studies; and additional mechanisms may be needed to be taken into account, to better reflect IgG clearance and subsequent dynamics at these later timepoints.

### Model sensitivity analysis

Local and global sensitivity analyses were performed for the immune activation parameters (*k9, k12, k13, kmat_3, Vmax*), to identify and assess parameters with the highest influence on ASC kinetics and the overall strength of the B cell response. Simulations were run over a 350-day period, for four variables: ASC counts in blood; bone marrow; lymph nodes; and spleen.

**Figure 4:**
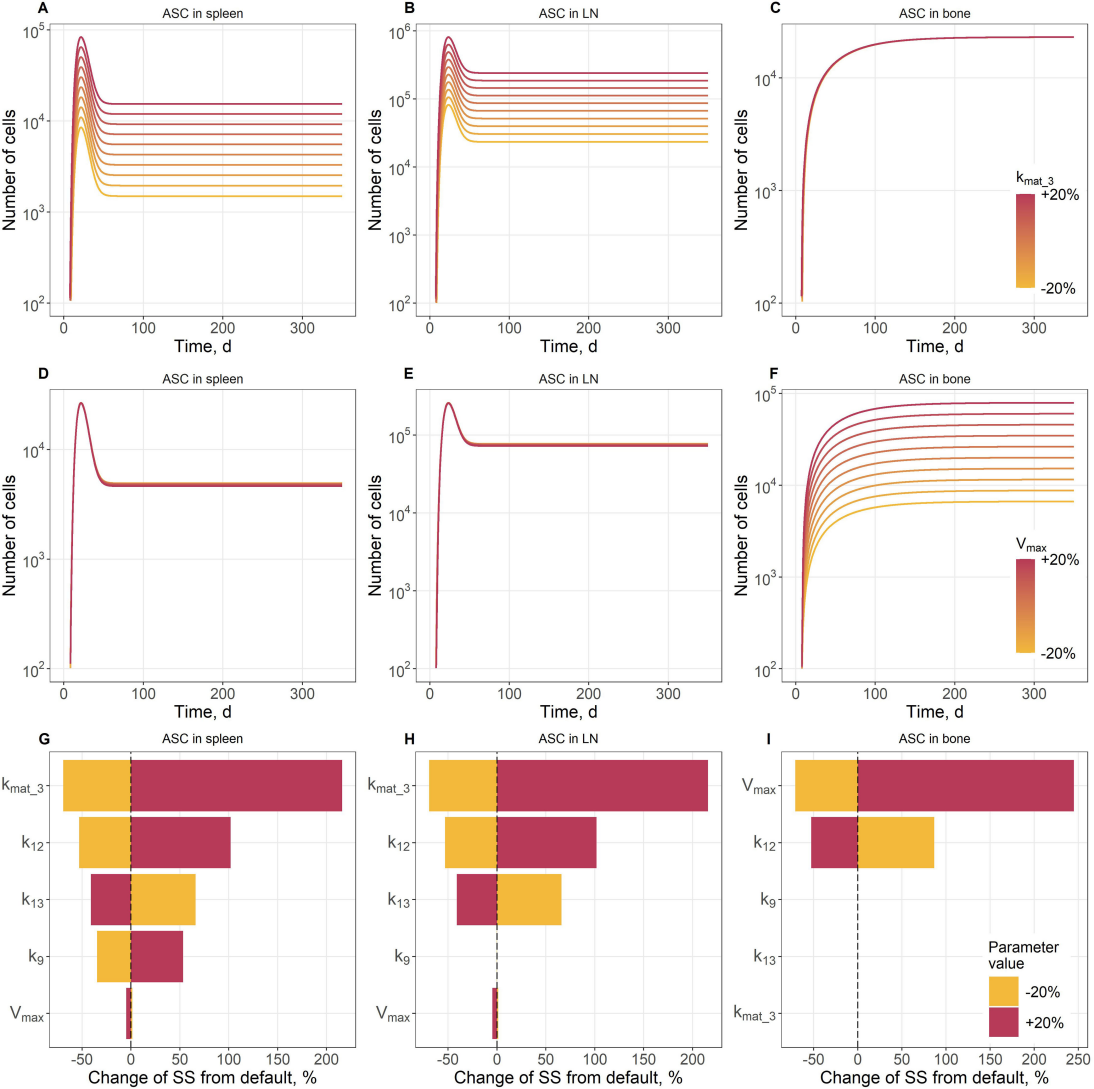
Local sensitivity analysis for ASC dynamics. Local sensitivity analysis for ASC dynamics in the spleen **(A,D,G)**, lymph nodes **(B,E,H)**, and bone **(C,F,I)**. **(A-F)** Family of curves for the *kmat_3* **(A-C)** and *Vmax* **(D-F)** parameters. Red curves are for large values of these parameters, yellow curves are for low values. **(G-I)** Tornado plots for ASC steady-state numbers. Red bars: +20% to the default of the parameter; yellow bars: -20% to the default of the parameter.

For the local sensitivity analysis, a family of curves was generated by varying each parameter within ±20% of its estimated value, resulting in 10 curves per parameter (**Figures 4A-F**). Additionally, tornado plots were displayed, to illustrate the sensitivity of both the maximum cell count and the steady-state cell count at Day 200 post-immunization. Parameter variations for the tornado plot were also set at ±20% of the estimated values (**Figure 4**). The analysis focused on two key parameters: *kmat_3* (**Figures 4A-C**) and *Vmax* (**Figures 4D-F**), with results shown for ASC populations in the spleen (**Figures 4A-D**), lymph nodes (**Figures 4B-E**), and the bone marrow (**Figures 4C-F**) - the tissues most relevant to the immune response and long-term ASC survival. Complete local sensitivity analysis results showing the effect of each parameter are provided in **Supplementary Figure 5**.

The results indicate that ASC behavior in the spleen and lymph nodes was strongly dependent on the maturation rate constant which governs the generation of ASC (**Figures 4A, B**). However, this parameter had little-to-no effect on ASC dynamics in the bone marrow (**Figure 4C**). In contrast, *Vmax*, which represents the size of the immune niche for ASC survival in the bone marrow, exerted the strongest influence on ASC concentrations in the bone marrow (**Figure 4F**), with minimal impact on spleen and lymph node dynamics (**Figures 4D, E**). In addition, a one-at-a-time sensitivity analysis was conducted to examine how individual parameter variations would affect steady-state levels of ASC (**Figures 4G-I**). Although the analysis was performed for all calibrated parameters and all variables, only results for ASC levels in the spleen (**Figure 4G**), lymph nodes (**Figure 4H**), and bone marrow (**Figure 4I**) are presented here. The analysis revealed that increasing the ASC maturation rate, *kmat_3*, by 20% nearly doubled ASC levels in both the spleen and lymph nodes, whereas a 20% decrease in this parameter led to a ~50% reduction. *Vmax* likely reflects the size and availability of survival niches for plasma cells within the tissue. Interestingly, the second most sensitive parameter across all three tissues was *k12*, the rate constant governing the return of ASC from the bone marrow to the blood. This suggests that ASC recycling or egress from the bone marrow may play a critical role in shaping the overall distribution and maintenance of ASC populations throughout the immune system. The observed ultrasensitive responses were likely related to the nonlinear structure of the model equations, including the saturable transport kinetics and compartmental coupling between ASC generation, migration, and niche-limited survival. The complete one-at-a-time sensitivity analysis results are available in **Supplementary Figure 6**.

A global sensitivity analysis was also conducted for the same variables and parameters using the eFAST method (71). The analysis was performed for both maximal cell counts and at quasi steady-state on Day 200. Parameters were independently sampled from uniform distributions within ±20% of the estimated values. An analysis was performed with a sample size of 1000 (**Supplementary Figure 7**). Results were consistent with the local sensitivity assessment, reinforcing the identification of key parameters that govern ASC dynamics across different tissues. The total-order sensitivity indices for the ASC maturation rate parameter, *kmat_3*, were higher than the first-order indices in the spleen, blood, and lymph nodes. This indicates that the impact of *kmat_3* on ASC levels in these tissues is not limited to a direct effect but also involves significant interactions with other parameters and variables, suggesting interdependence of ASC generation and distribution across tissues. In contrast, for the bone marrow compartment, the first-order and total-order sensitivity indices for the parameter *Vmax* were nearly identical, meaning that ASC dynamics in the bone marrow are predominantly governed by intrinsic properties within this compartment, with minimal influence from cross-compartmental interactions.

### Exploration of ASC dynamics variability

**Figure 5:**
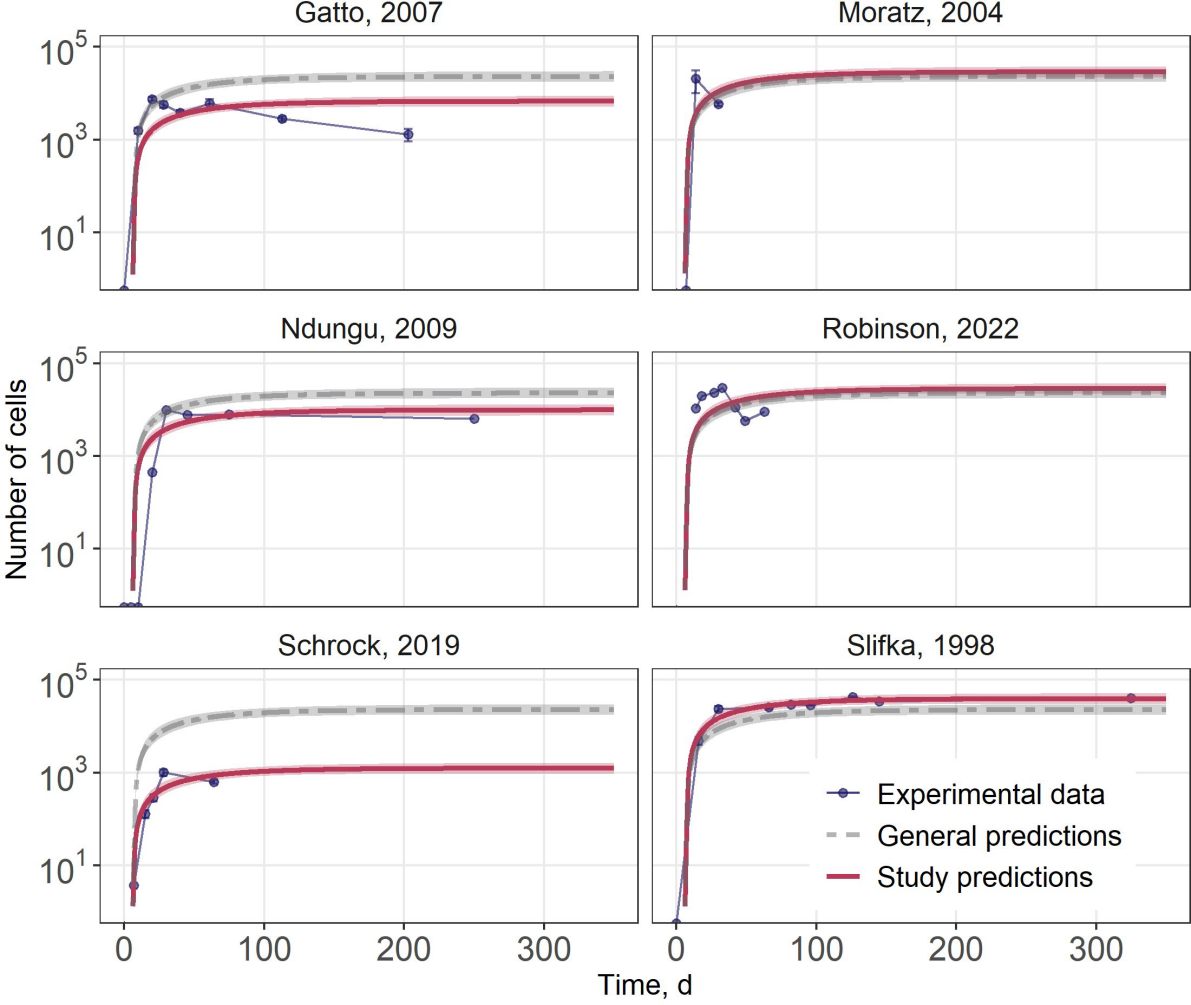
Analysis of inter-study heterogeneity. Predictions of ASC dynamics in the bone marrow across various experimental studies. Red line: individual predictions with 95% confidence intervals for each study; gray dashed line: general predictions with 95% confidence intervals; blue lines with points: experimental data. Error bars reflect the variability measure reported in the original publications.

In order to explore potential factors causing inter-study variability in ASC dynamics, simulations of the ASC response were performed with varying the *Vmax* parameter, which represents the compartment’s capacity for ASC accumulation and survival (**Figure 5**). For this purpose, each experimental dataset was fitted separately while estimating only the *Vmax* parameter, whereas all other parameters were fixed to the values obtained in the global calibration. All individual fits successfully converged, and the RSE of the estimated *Vmax* values remained below 12%, indicating stable parameter estimation. Across the six datasets, the estimated *Vmax* values ranged from 26.66 to 836 cells/day, with 492.8 cells/day representing the typical calibrated value. The lowest value was specifically selected to capture the markedly reduced ASC levels observed in the study by Schrock et al. (37), where ASC counts were consistently an order of magnitude lower, as compared to other datasets.

These simulations demonstrated that adjusting a single parameter, Vmax, representing the maximum capacity of survival niches in the bone marrow, may be sufficient to capture a significant portion of the variability observed in experimental studies. This observation suggests that the observed variability in bone marrow ASC levels may be predominantly explained by differences in the size or number of survival niches available for long-lived plasma cells in the bone marrow. Such structural heterogeneity across animals or experimental conditions could represent a key source of biological variability, and the model provided a mechanistic framework for capturing and interpreting this variation.

## Discussion

The application of mathematical modeling and theoretical concepts has positively influenced multiple aspects of immunological research (72–75). While numerous mathematical models have been developed to describe T cell homeostasis, including studies of T cell dynamics and multi-compartmental modeling of T cell migration (72, 73, 76–78), such comprehensive theoretical studies are indeed still limited in the context of B cell immunity (30). Several mathematical models have been developed previously, to describe intracellular signaling processes following B cell receptor activation and the transcriptional regulation underlying B cell fate decisions, e.g., models by Martínez et al. and Roy et al., which studied regulatory networks involving transcription factors such as BCL6, IRF4, and BLIMP1, which control activation of B cells and differentiation into plasma cells (79–82). Several systems biology models have described bistable gene regulatory networks involving transcription factors that control the all-or-none transition from naïve B cells to plasma cells. These studies demonstrated that environmental perturbations, including aryl hydrocarbon receptor agonists such as TCDD, can modulate the switching threshold and probabilistic nature of differentiation by altering regulatory pathway activity (83–85). In parallel, there have been modeling efforts focused on B cell development and the maturation of specific subpopulations, e.g., the model by Garnett et al. describing the dynamics of immature B cell subsets in the bone marrow under homeostatic regulation by mature B cells (86). Also, models have been developed to dynamically describe antigen-driven responses; for example, Ganusov et al. presented a model featuring the generation of ASC following vaccination (87). Similarly, Vicini and colleagues developed a mechanistic model capturing the emergence of plasma cell and therapeutic protein immunogenicity (88, 89). Additionally, there are models focused on infections; Lee et al. published a model describing B cell activation, clonal expansion, and antibody secretion in response to influenza A viral infection (90). More recently, a quantitative systems pharmacology model of B cell differentiation was developed to investigate determinants of response to rituximab-mediated B cell depletion in autoimmune diseases. This model integrated multiple B cell maturation stages across tissues and incorporated pharmacokinetic and pharmacodynamic processes associated with anti-CD20 therapy (91). These approaches have provided important quantitative context for studying therapeutic perturbations of the B cell system, with a primary focus on drug-driven depletion dynamics rather than antigen-induced humoral immune responses. While these models provide valuable mechanistic insights into specific stages of B cell biology, starting from development in the bone marrow to antigen-driven differentiation in peripheral lymphoid tissues, they fall short in capturing the intercompartmental dynamics of B cell subpopulations across tissues. Specifically, most models lack a joint structure that integrates activation, differentiation and migration of B cells from the bone marrow to secondary lymphoid organs, and finally to effector niches such as the bone marrow, the gastrointestinal tract, or the lungs. This limitation hinders the ability to fully understand tissue-specific contribution to systemic humoral immunity, particularly under pathological conditions such as autoimmunity or chronic inflammation; yet these processes represent essential elements at the core of any quantitative systems pharmacology application of B cell immunology, specifically in the context of translational studies (48, 92, 93).

The mechanistic model presented here provided an integrated framework to describe B cell and ASC lifecycle and dynamics in mouse, with physiologically relevant distribution across tissues. By incorporating key biological processes from the generation of immature B cells in the bone marrow, maturation, migration to secondary lymphoid organs and activation upon antigen exposure, the model simultaneously captured B cell homeostasis and the dynamic T cell-dependent response following antigen exposure. Its multi-compartmental structure adequately represents the physiological routes and niches that B cells and ASC adopt during an immune response, offering a novel perspective on their behavior and interactions. The model explicitly included migratory pathways across the bone marrow, the spleen, lymph nodes and peripheral tissues, enabling a systems-level understanding of how ASC populations are generated, maintained, and distributed. This approach contrasts with most previously published models that focused on single compartments or on limited subsets of the B cell lineage, thus providing a more holistic view of B cell biology and adaptive immunity (30).

The sequential modeling strategy allowed to consider various types of quantitative experimental information, encompassing multiple B cell subpopulations and ASC dynamics across different tissues. This stepwise approach not only ensured the robustness of parameter estimates but also enhanced the biological interpretability of model components (94–96). Importantly, the model successfully reproduced the steady-state homeostasis of naive B cells as well as the complex dynamics of B cell immune response, consistent with previously published experimental observations on ASC behavior following immunization (87, 97). Furthermore, parameter estimation robustness was thoroughly evaluated using multiple tools, including evaluation of the variance-covariance matrix, multi-start fitting and likelihood profiling analyses, in accordance with modeling and simulation guidelines in effect in the pharmaceutical industry (31, 98–100), thereby ensuring reproducibility and potential for further applications of the proposed model in an industrial R&D setting (101, 102). In particular, the prediction power of the model was tested via external validation, to test the ability of the model to adequately describe independent data which had not been used in model calibration steps (103). Model predictions of antigen-specific IgG antibody kinetics were evaluated using independent datasets following mouse immunization, with no antibody data included in the calibration process (34–36, 67, 68). During this validation phase, the model adequately reproduced the dynamics of IgG concentrations, with an average prediction error of approximately 25%. Such an agreement between predicted and observed profiles supported the model’s validity in performing predictions and its ability to extrapolate beyond the primary calibration targets, namely ASC counts. The validation process highlighted certain simplifications in the antibody submodel. In the current model formulation, antibody production is represented by a constant secretion rate per ASC, together with a time-invariant IgG clearance rate. While this formulation adequately described the overall relationship between ASC dynamics and antibody levels, experimental evidence suggests that long-lived plasma cells may undergo functional adaptation over time, potentially altering per-cell antibody production. In addition, long-term IgG homeostasis may be influenced by processes such as FcRn-mediated recycling and competition for survival niches (104–106). It should also be noted that the IgG datasets compiled for validation originated from multiple studies conducted under different experimental conditions, which likely introduced substantial between-study variability that is not explicitly represented in the present framework. Incorporating time-dependent secretion rates, adaptive clearance mechanisms, or explicit variability components may therefore improve the ability of the model to reproduce long-term antibody dynamics across studies.

Local sensitivity analysis revealed a tissue-specific partitioning of regulatory mechanisms: the ASC maturation rate constant (*kmat_3*) controlled ASC dynamics in secondary lymphoid organs, while the maximum capacity of survival niches (*Vmax*) primarily controlled ASC accumulation in the bone marrow. The ultrasensitive responses identified in the sensitivity analysis were not associated with parameter estimation instability. The robustness of the calibration procedure was confirmed using a multi-start optimization, where perturbations of an initial parameter guess within +/-50% resulted in optimized parameter values differing by less than 15%, while the -2 log-likelihood values remained essentially unchanged.

Furthermore, likelihood profiling demonstrated well-defined minima for all estimated parameters, supporting practical identifiability of the model structure. This dichotomy aligns well with known biological principles, with the generation and differentiation of ASC in secondary lymphoid organs depending mostly on antigenic stimulation and local cellular interactions, while the long-term maintenance of ASC and the humoral immune response being highly affected by the availability and size of specialized survival niches within the bone marrow microenvironment (7, 8). These niches provide critical trophic support for plasma cells, including signals mediated by stromal cells and cytokines such as APRIL and IL-6, enabling their longevity (51, 107).

Interestingly, the rate constant of ASC migration from bone to blood, *k12*, which represents the egress or recycling of ASC from the bone marrow back into the circulation, emerged as the second most influential parameter across all examined tissues. This suggests that ASC are not static entities but dynamically recirculate, potentially allowing redistribution or replenishment of peripheral pools. This insight resonates with emerging experimental evidence on plasma cell trafficking and has implications for understanding systemic immune regulation and memory maintenance (9, 108). This is consistent with the experimental data used for model calibration, which exhibited substantial variation in steady-state and in maximal cell counts. For example, our calibration data showed a mean of 1.6 * 10^4 (SE ± 2.1 * 10^4) in steady-state cell counts after 200 days, while the maximal number of ASC in the bone marrow reported by Slifka et al. (36) was nearly two orders of magnitude higher than that found by Schrock et al. (37).

Model-based exploratory simulations suggested that a considerable part of the observed variability could be captured by changes in a single parameter representing the capacity of survival niches in the bone marrow (Vmax). Allowing this parameter to vary across studies enabled the model to reproduce much of the variation in ASC accumulation observed in the compiled datasets. At the same time, it is likely that the full range of experimental observations across tissues and studies reflects the combined influence of multiple biological and experimental factors. Consequently, reproducing all study-specific dynamics in detail would probably require variation in several model parameters. Within the scope of the present work, we adopted a parsimonious approach and used variation in Vmax as a mechanistically interpretable factor that captured a substantial part of the inter-study differences in ASC dynamics. These findings suggest that differences in the size or availability of survival niches within the bone marrow microenvironment may represent an important biological contributor to variability in humoral immune responses. Such heterogeneity in bone marrow niches may be particularly relevant in the context of autoimmune diseases (109, 110), whereby dysregulated plasma cell survival may contribute to chronic antibody-mediated pathology (12–14). Intrinsic factors - e.g., genetic predisposition, age-related changes in immune architecture, or a history of repeated immune activation - may influence the availability and stability of survival niches for autoreactive plasma cells (111, 112). In addition, inflammatory signals and tissue remodeling, commonly observed in autoimmune settings, could further shape the bone marrow microenvironment (49, 113). While the present model does not explicitly address autoimmune pathology, its structure provides a solid foundation for future extensions aimed at exploring how microenvironmental variability may affect the persistence of autoreactive ASCs. Furthermore, these results emphasize the pivotal role of the bone marrow as a regulator of long-term plasma cell maintenance and, by extension, durable humoral immunity (10, 114). It also suggests potential avenues for novel therapeutic manipulation, for instance, by targeting niche availability or functionality to stop antibody production in autoimmune diseases (115).

While the present model demonstrated robust performance and provided valuable mechanistic insights, several limitations should be considered. In the current model, lymph nodes were represented as a single compartment describing the aggregate ASC population across all lymph nodes. This assumption corresponds to a homogeneous distribution of ASC among individual nodes. In reality, lymph nodes differ in size, drainage, and immune cell phenotype distributions, etc. (116, 117), which may lead to variability in local cell counts. However, quantitative data enabling node-specific parameterization of ASC dynamics remain limited. Within the present model structure, such heterogeneity would primarily result in a redistribution of cells among individual nodes, while having a limited impact on the total lymph node ASC pool. Additionally, in the present model, systems-level ASC dynamics drove the overall effect, i.e. the above assumption should not affect the conclusions. However, extrapolation of the current modeling framework and translation of the model to clinical settings will require a concerted consideration of the lymphatic system network. In addition, activated B cell proliferation and differentiation into ASC are represented implicitly through an effective ASC generation rate. This formulation captures the aggregated processes of activation, clonal expansion and terminal differentiation, without explicitly modeling intermediate proliferative stages. While inclusion of dedicated proliferating B cell compartments could provide a more mechanistic description, the currently available experimental data are insufficient to support reliable parameterization of such processes. Introducing this level of detail would therefore increase model complexity and parameter uncertainty, without substantially altering the qualitative behavior or predictive performance of the model.

The model was calibrated using aggregated experimental data representing mean ASC dynamics across studies rather than individual trajectories. While the structure can mathematically reproduce single-study behavior, the calibrated parameters reflected systems-level averages. Differences between model predictions and individual datasets largely arise from inter-study variability and biological heterogeneity. Accounting for this explicitly would require a Bayesian hierarchical QSP approach, which could be considered in future developments of the current model. In this context, the time persistence of ASC predicted by the model reflects the presence of detectable ASC populations in secondary lymphoid organs reported in experimental studies, which were captured during model calibration (33, 34, 36).

The model currently lacks an explicit representation of regulatory feedback mechanisms and microenvironmental signaling that govern B cell differentiation and plasma cell survival. For example, interactions with T follicular helper cells, cytokines, and competition for survival niches are known to critically influence ASC fate (10, 118, 119). Including this regulatory feedback loop would enable simulations of immune modulation by external stimuli, immunotherapies, or pathological conditions. Secondly, the current model does not explicitly incorporate cytokine-mediated regulation and feedback mechanisms that critically influence B cell activation, differentiation, and long-term ASC survival. Among such factors, type I interferons (IFN-I) are known to modulate B cell responses, promote activation, and influence antibody production, particularly under pathological conditions such as systemic lupus erythematosus (SLE) (120). Recent systems pharmacology modeling of IFN-I-driven inflammation in SLE demonstrated how variations in IFN gene signature can profoundly affect therapeutic outcomes and immune cell dynamics in SLE (121). Incorporating IFN-I signaling into the model would improve its ability to capture pathological B cell responses characteristic of autoimmune diseases.

The antigenic stimulus is currently represented in a simplified and transient manner, consistent with an acute primary immune response. In contrast, autoimmune diseases are characterized by chronic or recurrent exposure to self-antigens, often associated with defective clearance mechanisms and sustained germinal center activity. Future iterations of the model would therefore require recalibration using experimental data from autoimmune-prone mouse strains, such as lupus models, to capture dysregulated B cell activation, prolonged germinal center reactions, and altered plasma cell survival dynamics. In parallel, the antigen module would be reformulated to represent persistent or self-renewing autoantigen exposure rather than a time-limited exogenous antigen pulse. Such modifications will allow the framework to transition from modeling a generic primary IgG response toward mechanistic simulations of autoreactive IgG production and chronic humoral autoimmunity.

Finally, explicit representation of autoreactive B cell selection thresholds, tolerance breakdown, and memory B cell dysregulation would further enhance the model’s relevance for autoimmune disease research and therapeutic development.

## Conclusions

The presented model accurately reproduced the steady-state distribution of B cell subpopulations across blood, the spleen, lymph nodes and the bone marrow, as well as the complex dynamics of ASC generation and long-term maintenance following antigenic stimulation. Sensitivity analyses revealed that ASC generation in secondary lymphoid organs was primarily governed by the maturation rate, whereas ASC accumulation and long-term maintenance in the bone marrow were predominantly influenced by the survival niche capacity. Simulations varying the size of the survival niche in the bone marrow captured the experimentally observed inter-study variability, suggesting that heterogeneity may be a key source of biological variation. Adapting the model to human immunological data and integrating disease-specific parameters will further enable its application in predicting treatment outcomes, understanding inter-patient variability, and guiding personalized therapeutic strategies in the context of autoimmune diseases.

## Data Availability

The original contributions presented in the study are included in the article/supplementary material. Further inquiries can be directed to the corresponding authors.
