# Supplementary material for: Quantitative systems pharmacology model of B cell immune response in mouse
Source: Front Immunol. 2026 Apr 30;17:1745710. doi: 10.3389/fimmu.2026.1745710 (PMC13171755; doi:10.3389/fimmu.2026.1745710)
Supplement: Supplementary file 1 [file DataSheet1.pdf]

Supplementary Material

Quantitative systems pharmacology model of B cell immune response in mouse

Yaroslav Ugolkov[1,2,3], Alina Volkova[2,3], Gabriel Helmlinger[5], Kirill Peskov[2,3,4], Victor Sokolov[2,3]

[1] Faculty of Bioengineering and Bioinformatics, Lomonosov Moscow State University, Moscow, Russian Federation, [2] Marchuk Institute of Numerical Mathematics, Moscow, Russian Federation, [3] M&S Decisions LLC, Dubai, UAE,[4] Research Center of Model-Informed Drug Development, I.M. Sechenov First Moscow State Medical University, Moscow, Russian Federation, [5] Quantitative Medicines, Lexington, MA, United States

Corresponding Author: Yaroslav Ugolkov, yaroslav.ugolkov@msdecisions.tech

Supplementary Figures

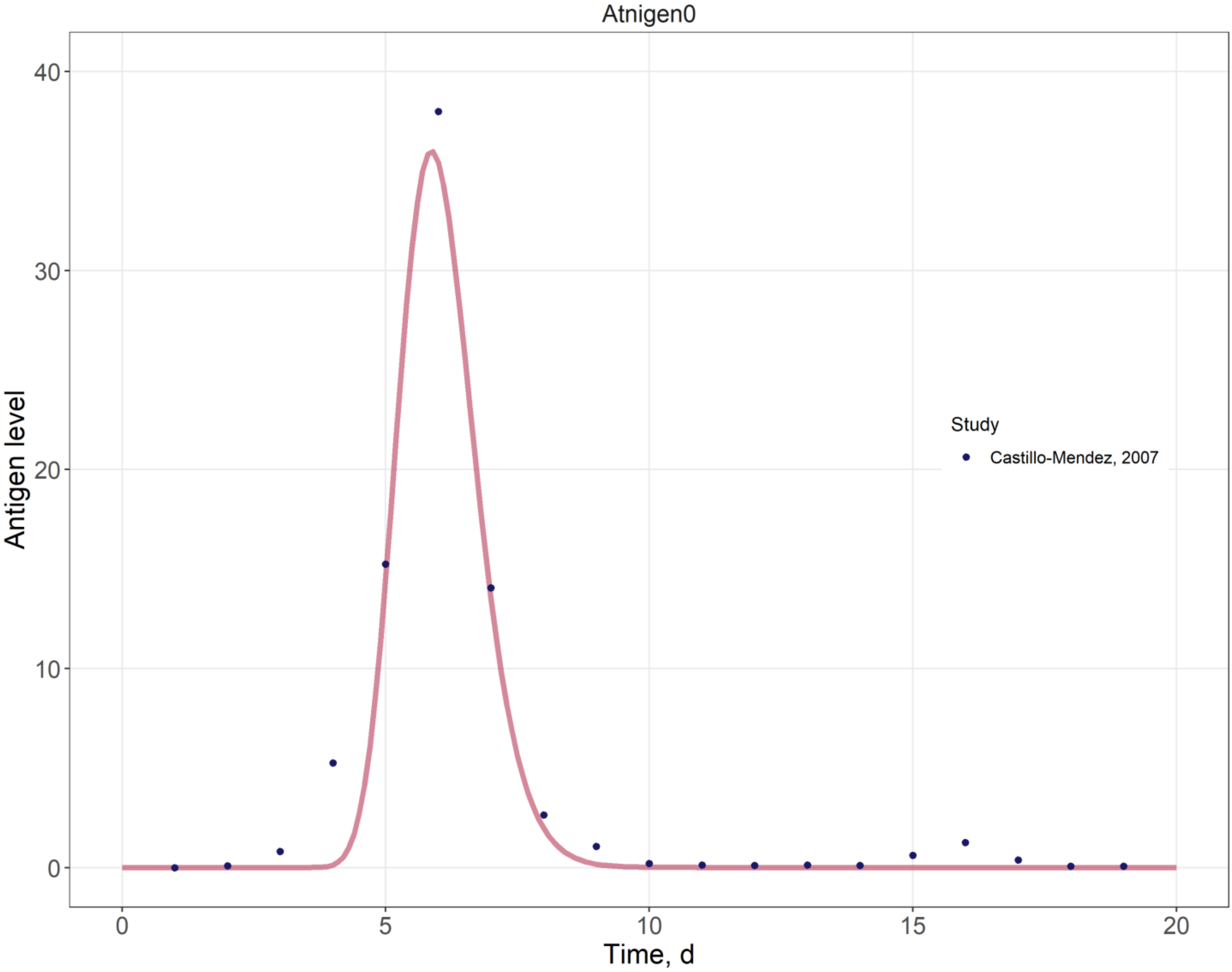

Figure S1. Model simulation of antigen dynamics.

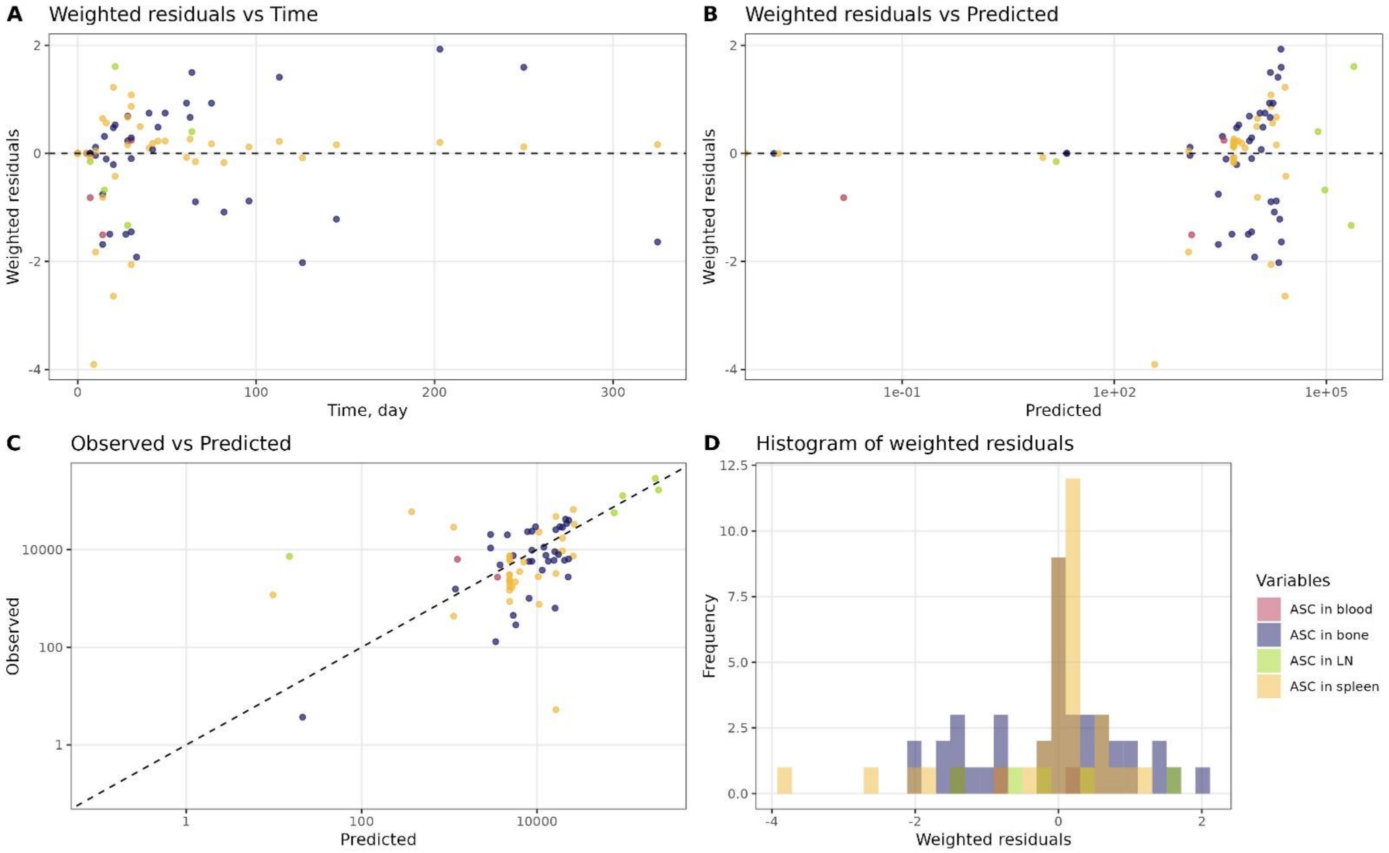

Figure S2. Model goodness-of-fit plots.

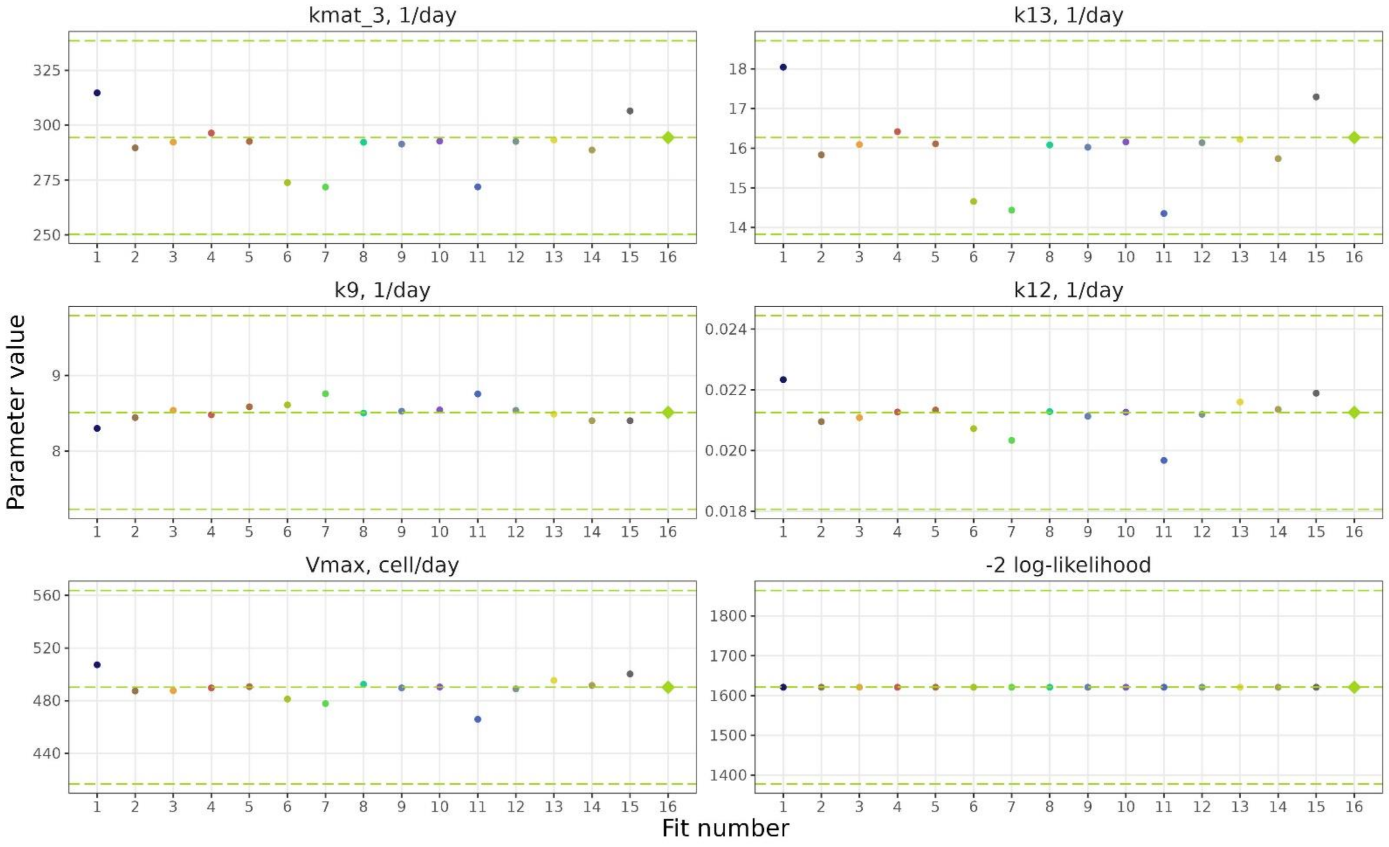

Figure S3. Multistart fitting.

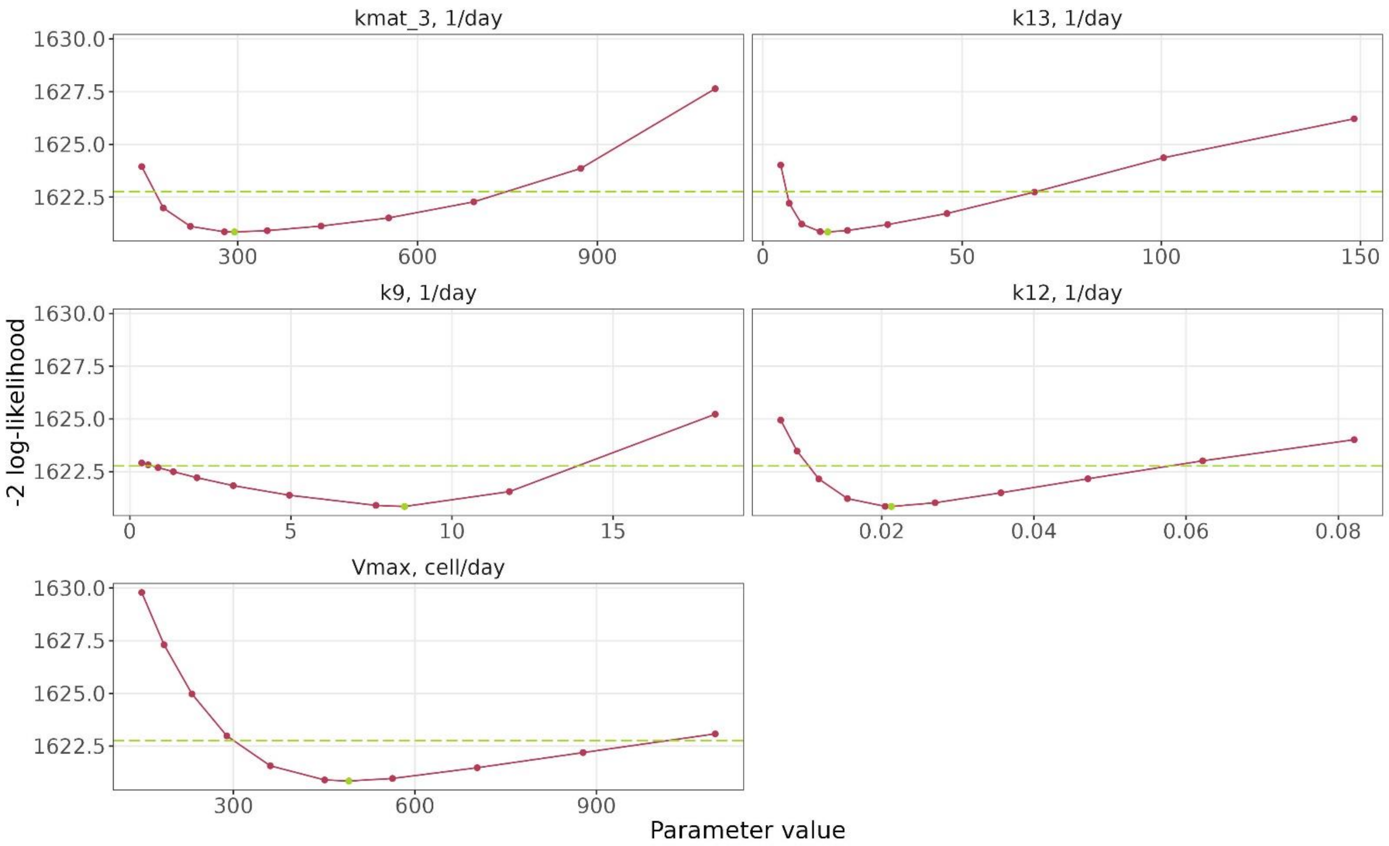

Figure S4. Likelihood profiling.

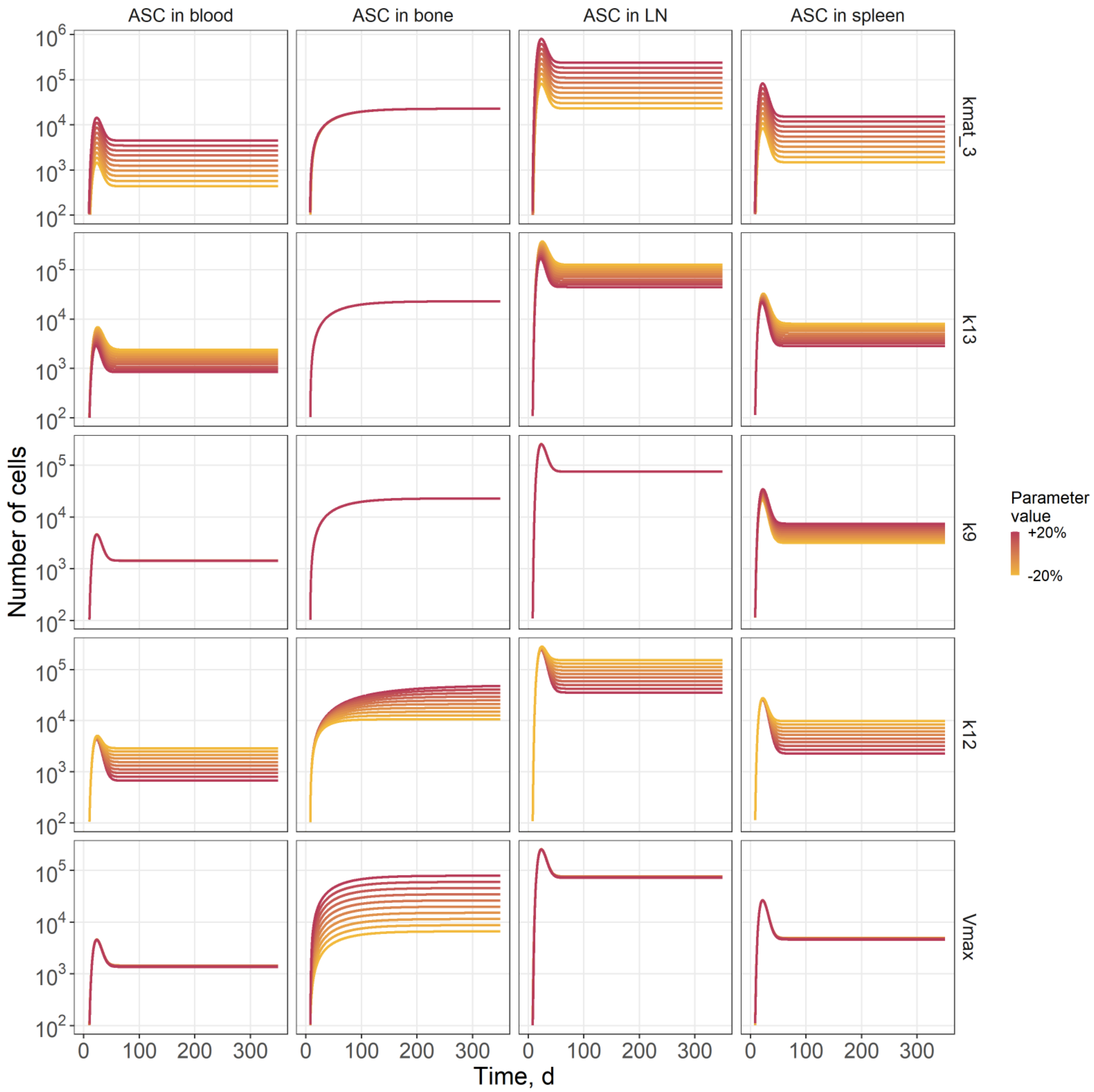

Figure S5. Full local sensitivity analysis using families of curves.

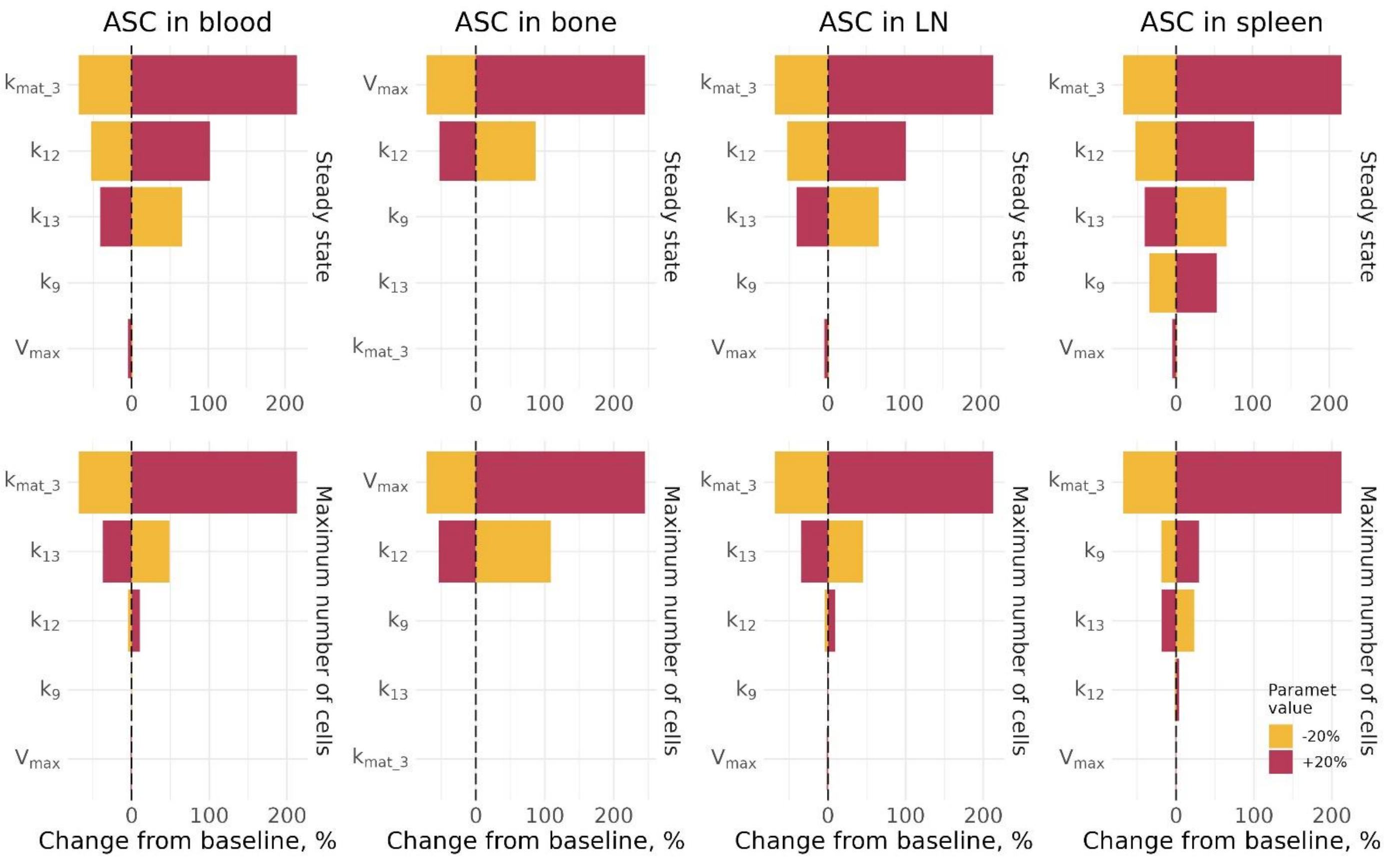

Figure S6. Full local sensitivity analysis using tornado plots.

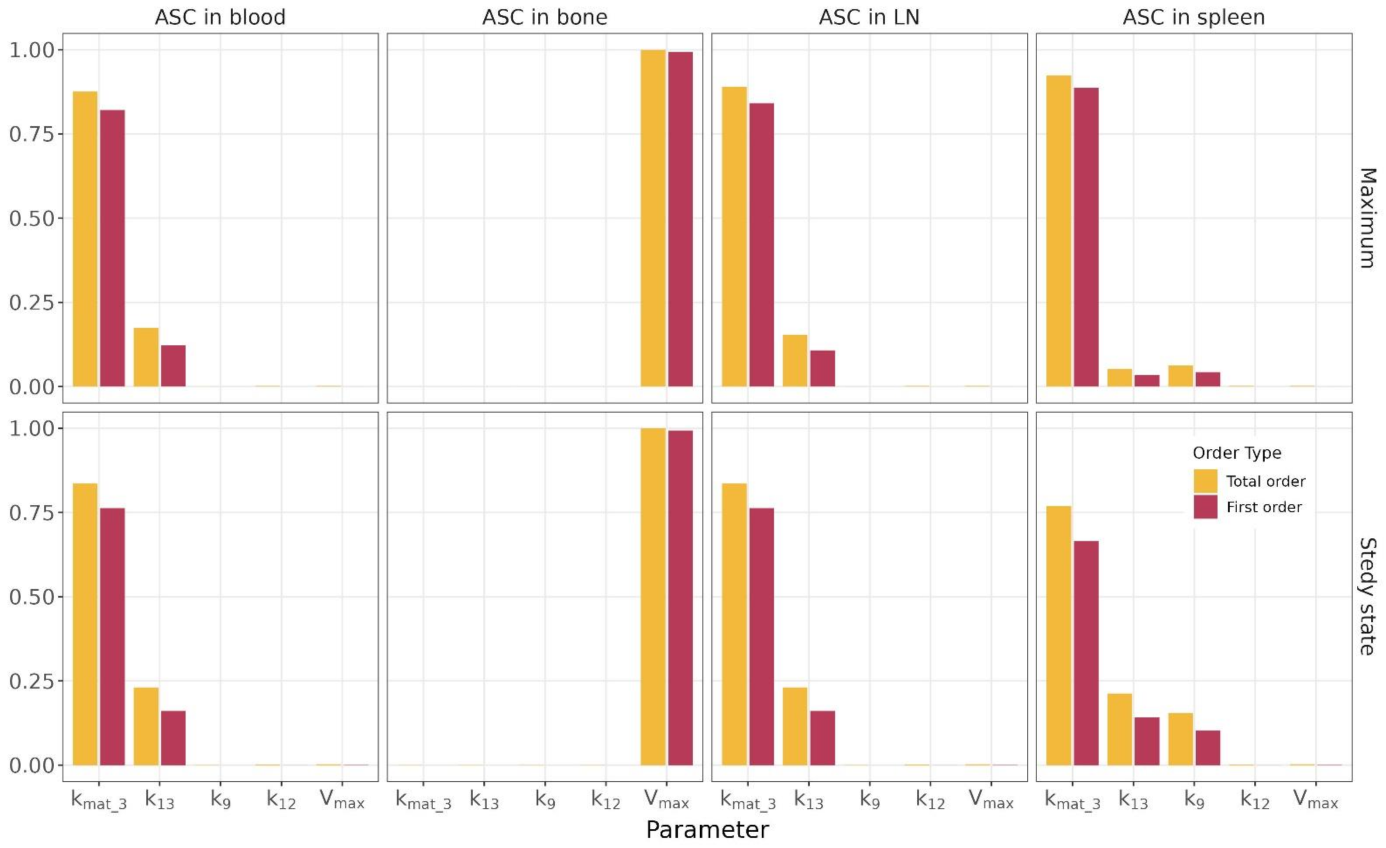

Figure S7. Global sensitivity analysis using eFAST

Supplementary Equations

$$kmat_1 = \frac{ksyn_{imm}}{ImmBone_{ss}}$$ Equation 1

$$k1 = \frac{kmat_1 * ImmBone_{ss}}{T1Bone_{ss}}$$ Equation 2

$$k3 = \frac{k1 * T1Bone_{ss} + k2 * T1Spleen_{ss}}{T1Blood_{ss}}$$ Equation 3

$$kmat_2 = \frac{k3 * T1Blood_{ss} - k2 * T1Spleen_{ss}}{T1Spleen_{ss}}$$ Equation 4

$$k7 = \frac{k6 * NaiveBlood_{ss} - deg_{ln} * NaiveSpleen_{ss}}{NaiveLN_{ss}}$$ Equation 5

$$k5 = \frac{k7 * NaiveLN_{ss} - k6 * NaiveBlood_{ss} + k4 * NaiveSpleen_{ss}}{NaiveBlood_{ss}}$$ Equation 6

$$kdeg_{nai} = \frac{kmat_2 * T1Spleen_{ss} - k4 * NaiveSpleen_{ss} + k5 * NaiveBlood_{ss}}{NaiveSpleen_{ss}}$$ Equation 7

$$\frac{dImmBone}{dt} = ksyn_{imm} - kmat_1 * ImmBone$$ Equation 8

$$\frac{dT1Bone}{dt} = kmat_1 * ImmBone - k1 * T1Bone$$ Equation 9

$$\frac{dT1Blood}{dt} = k1 * T1Bone + k2 * T1Spleen - k3 * T1Blood$$ Equation 10

$$\frac{dT1Spleen}{dt} = k3 * T1Blood - k2 * T1Spleen - kmat_2 * T1Spleen$$ Equation 11

$$\frac{dNaiveBlood}{dt} = k4 * NaiveSpleen - k5 * NaiveBlood + k7 * NaiveLN - k6 * NaiveBlood$$ Equation 12

$$\frac{dNaiveSpleen}{dt} = kmat_2 * T1Spleen - kdeg_{spl} * NaiveSpleen - k4 * NaiveSpleen + k5 * NaiveBlood$$ Equation 13

$$\frac{dNaiveLN}{dt} = k6 * NaiveBlood - k7 * NaiveLN - deg_{ln} * NaiveLN$$ Equation 14

$$\frac{dAntigen0}{dt} = \left(\frac{393.12}{t^2} - \frac{66.98}{t}\right) * \exp\left(189.1 - \frac{393.12}{t} - 66.98 * \ln(t)\right)$$ Equation 15

$$\frac{dAntigen1}{dt} = klag * Antigen0 - klag * Antigen1$$ Equation 16

$$\frac{dAntigen2}{dt} = klag * Antigen1 - klag * Antigen2$$ Equation 17

$$\frac{dAntigen3}{dt} = klag * Antigen2 - klag * Antigen3$$ Equation 18

$$\frac{dAntigen4}{dt} = klag * Antigen3 - klag * Antigen4$$ Equation 19

$$\frac{dAntigen5}{dt} = klag * Antigen4 - klag * Antigen5$$ Equation 20

$$\frac{dAntigen}{dt} = klag * Antigen5 - klag * Antigen$$ Equation 21

$$\frac{dASCSpleen}{dt} = kmat_3 * Antigen * \frac{NaiveSpleen}{10^6} - k8 * ASCSpleen + k9 * ASCBlood$$

Equation 22

$$\frac{dASCLN}{dt} = kmat_3 * Antigen * \frac{NaiveLN * 22}{10^6} - k11 * ASCLN + k10 * ASCBlood$$

Equation 23

$$\frac{dASCBone}{dt} = \frac{Vmax * ASCBlood}{Khalf + ASCBlood} - k12 * ASCBone$$

Equation 24

$$\frac{dASCBlood}{dt} = k11 * ASCLN + k8 * ASCSpleen + k12 * ASCBone + k14 * ASCPeripheral - \left(k9 + k10 + k13 + \frac{Vmax}{Khalf + ASCBlood}\right) * ASCBlood$$

Equation 25

$$\frac{dASCPeripheral}{dt} = k13 * ASCBlood - k14 * ASCPeripheral$$

Equation 26

$$ASCTotal = ASCSpleen + ASCBone + ASCBlood + ASCLN$$

Equation 27

$$\frac{dIgGBlood}{dt} = ksyn_{igg} * ASCTotal - kdeg_{igg} * IgGBlood$$

Equation 28

Supplementary Tables

Table S1. Summary of publication used for model development, calibration, and validation.

Reference	Measured variables	Mouse strain	Sex	Age (weeks)	Immunogen / antigen	Antigen class	Administration route	Use in model development
Brennecke, 2018 (1)	T1 B cell in the blood	BALB/c	-	8–14	Tamoxifen	Experimental compound	Oral	Steady-state calibration
Bucheli, 2024 (2)	IgG in blood	BALB/cJRj	Female	7–10	Tetanus toxin heavy chain fragment	Protein antigen	Intraperitoneal (i.p.)	Parameter calculation
Castillo-Mendez, 2007 (3)	antigen in blood	C57BL/6	Female	6–8	Plasmodium chabaudi	Parasite	Intraperitoneal (i.p.)	Antigen function calculation
Jones, 2015 (4)	Follicular Naive B cell	C57BL/6	-	-	NP18-chicken γ-globulin	Hapten–protein antigen	Intraperitoneal (i.p.)	Parameter calculation
Dejbakhsh-Jones, 1995 (5)	Immature B cell in bone	BALB/c	Male	6–12	-	-	-	Parameter calculation
Gatto, 2007 (6)	ASC in spleen, bone; IgG in blood	C57BL/6	-	-	Virus-like particles (VLP-Q)	Virus-like particle	Intravenous (i.v.)	ASC model calibration and validation
Jones, 2016 (7)	immature B cells in bone; T1 B cell in bone	C57BL/6	Both	5–8	Chicken ovalbumin	Protein antigen	Intraperitoneal (i.p.)	Steady-state calibration
Loder, 1999 (8)	Naive B cell in the blood	C57BL/6, CBA/J, and CBA/N	-	6–8	-	-	-	Steady-state calibration
Mackay, 1999 (9)	Naive B cell in lymph node	C57BL/6	-	-	-	-	-	Steady-state calibration
Maqueda-Alfaro, 2021 (10)	IgG in blood	BALB/c	Male	6–8	Dengue virus 2	Virus	Intradermal (i.d.)	Model validation
Moratz, 2004 (11)	ASC in spleen, bone, blood; IgG in blood	C57BL/6J	-	6–16	Sheep red blood cells	Cellular antigen	-	ASC model calibration and validation
Moreno, 2004 (12)	IgG in blood	BALB/c	Female	8–9	7-valent Pnc conjugate vaccine	Vaccine	Intraperitoneal (i.p.)	Model validation

Ndungu, 2009 (13)	ASC in spleen, bone	C57BL/6	Female	6–12	Plasmodium chabaudi	Parasite	Intraperitoneal (i.p.)	ASC model calibration
Pellegrini, 2007 (14)	Naive B cell in the spleen; T1 B cell in the spleen	C57BL/6	Female	6–8	Trypanosoma cruzi	Parasite	Intradermal (i.d.)	Steady-state calibration
Petkova, 2008 (15)	Naive B cell in the blood	C57BL, BALB/c	Both	24	-	-	-	Steady-state calibration
Robinson, 2022 (16)	ASC in spleen, bone	C57BL/6	-	6–8	Protein keyhole limpet hemocyanin	Protein antigen	Intraperitoneal (i.p.)	ASC model calibration
Schrock, 2019 (17)	ASC in bone, lymph node	C57BL/6J	-	-	Np-ova/cfa	Hapten–protein antigen	Intradermal (i.d.)	ASC model calibration
Slifka, 1998 (18)	ASC in spleen, bone; IgG in blood	BALB/c	-	5–8	LCMV	Virus	Intraperitoneal (i.p.)	ASC model calibration and validation
Slifka, 1998 (19)	ASC in spleen, bone; IgG in blood	BALB/c	-	-	LCMV	Virus	Intraperitoneal (i.p.)	Model structure development
Verheijen, 2020 (20)	Follicular mature and germinal center B cells in blood, spleen, lymph node	C57Bl6/J	Female	8–12	Tamxoxifen	Experimental compound	Intraperitoneal (i.p.)	Parameter calculation
Vieira, 1988 (21)	IgG in blood	C57Bl6/J	Female	-	Purified protein	Protein antigen	Intraperitoneal (i.p.)	Parameter calculation

Supplementary RxODE model code

```
{
ImmBone_ss = 1.97  #cell / 10^6
T1Bone_ss = 0.76  #cell / 10^6
T1Blood_ss = 0.004  #cell / 10^6
T1Spleen_ss = 4.40  #cell / 10^6
NaiveSpleen_ss = 24.30  #cell / 10^6
NaiveBlood_ss = 4.64  #cell / 10^6
NaiveLN_ss = 1.90  #cell / 10^6

k2 = 1.01 #1/day
k4 = 10.08 #1/day
k6 = 4.9 #1/day

ksyn_imm = 20  #(cell / 10^6)/day
kdeg_ln = 0.01 #1/day

k8 = 2.52 #1/day
k9 = 8.53 #1/day
k10 = 143.6 #1/day
k11 = 2.72 #1/day
k12 = 0.02 #1/day
k13 = 16.26 #1/day
k14 = 0.02 #1/day

ksin_igg = 22 #pg/cell/day
kdeg_igg = 0.12 #1/day
kmat_3 = 294.19 #1/day

Vmax = 490.6 #cell/day
Khalf = 0.1 #cell

MTT =18
```

```
klag =7/ MTT



kmat_1 = (ksyn_imm)/(ImmBone_ss )
k1 = (kmat_1* ImmBone_ss)/(T1Bone_ss )
k3 = (k1 * T1Bone_ss+ k2 * T1Spleen_ss)/(T1Blood_ss )
kmat_2 = (k3 * T1Blood_ss- k2 * T1Spleen_ss)/(T1Spleen_ss )
k7 = (k6 * NaiveBlood_ss- kdeg_ln* NaiveSpleen)/(NaiveLN_ss )
k5 = (k7 * NaiveLN_ss- k6 * NaiveBlood_ss + k4 * NaiveSpleen_ss)/(NaiveBlood_ss )
kdeg_spl = (kmat_2* T1Spleen_ss- k4 * NaiveSpleen_ss+ k5 * NaiveBlood_ss)/(NaiveSpleen_ss )

ImmBone(0) = ImmBone_ss
T1Bone(0) = T1Bone_ss
T1Blood(0) = T1Blood_ss
T1Spleen(0) = T1Spleen_ss
NaiveBlood(0) = NaiveBlood_ss
NaiveSpleen(0) = NaiveSpleen_ss
NaiveLN(0) = NaiveLN_ss

Antigen0(0) = 0
Antigen1(0) = 0
Antigen2(0) = 0
Antigen3(0) = 0
Antigen4(0) = 0
Antigen5(0) = 0
Antigen(0) = 0
ASCSpleen(0) = 0
ASCLN(0) = 0
ASCBone(0) = 0
ASCBlood(0) = 0
ASCPeripheral(0) = 0
IgGBlood(0) = 0
```

```
d/dt(ImmBone) = ksyn_imm- kmat_1* ImmBone

d/dt(T1Bone) = kmat_1* ImmBone - k1 * T1Bone

d/dt(T1Blood) = k1 * T1Bone + k2 * T1Spleen - k3 * T1Blood

d/dt(T1Spleen) = k3 * T1Blood - k2 * T1Spleen - kmat_2* T1Spleen

d/dt(NaiveBlood) = k4 * NaiveSpleen - k5 * NaiveBlood + k7 * NaiveLN - k6 * NaiveBlood

d/dt(NaiveSpleen) = kmat_2* T1Spleen  - kdeg_spl* NaiveSpleen - k4 * NaiveSpleen + k5 * NaiveBlood

d/dt(NaiveLN) = k6 * NaiveBlood - k7 * NaiveLN- kdeg_ln* NaiveLN

d/dt(Antigen0) = (393.12 / t^2 - 66.98 / t)* exp( 189.1 - 393.12 / t -66.98 * log(t))

d/dt(Antigen1) = klag * Antigen0 - klag * Antigen1

d/dt(Antigen2) = klag * Antigen1 - klag * Antigen2

d/dt(Antigen3) = klag * Antigen2 - klag * Antigen3

d/dt(Antigen4) = klag * Antigen3 - klag * Antigen4

d/dt(Antigen5) = klag * Antigen4 - klag * Antigen5

d/dt(Antigen) = klag * Antigen5 - klag * Antigen

d/dt(ASCSpleen) = kmat_3* Antigen *  NaiveSpleen  - k8 * ASCSpleen + k9 * ASCBlood

d/dt(ASCLN) = kmat_3* Antigen *  (NaiveLN * 22)  - k11 * ASCLN + k10 * ASCBlood

d/dt(ASCBone) = (Vmax * ASCBlood)/(Khalf+ ASCBlood)- k12 * ASCBone
```

```
d/dt(ASCBlood) = k11 * ASCLN + k8 * ASCSpleen + k12 * ASCBone + k14 * ASCPeripheral - (k9 + k10 + k13 +Vmax/(Khalf + ASCBlood))* ASCBlood

d/dt(ASCPeripheral) = k13 * ASCBlood - k14 * ASCPeripheral

ASCTotal = ASCSpleen + ASCBone + ASCBlood + ASCLN

d/dt(IgGBlood) = ksin_igg* ASCTotal - kdeg_igg* IgGBlood
}
```

Supplementary references

1. Brennecke A-M, Düber S, Roy B, Thomsen I, Garbe AI, Klawonn F, Pabst O, Kretschmer K, Weiss S. Induced B Cell Development in Adult Mice. *Front Immunol* (2018) 9: doi: 10.3389/fimmu.2018.02483

2. Bucheli OTM, Rodrigues D, Portmann K, Linder A, Thoma M, Halin C, Eyer K. Single-B cell analysis correlates high-lactate secretion with stress and increased apoptosis. *Sci Rep* (2024) 14:8507. doi: 10.1038/s41598-024-58868-0

3. Castillo-Méndez SI, Zago CA, Sardinha LR, Freitas do Rosário AP, Alvarez JM, D'Império Lima MR. Characterization of the spleen B-cell compartment at the early and late blood-stage Plasmodium chabaudi malaria. *Scand J Immunol* (2007) 66:309–319. doi: 10.1111/j.1365-3083.2007.01972.x

4. Jones DD, Wilmore JR, Allman D. Cellular Dynamics of Memory B Cell Populations: IgM+ and IgG+ Memory B Cells Persist Indefinitely as Quiescent Cells. *J Immunol Baltim Md 1950* (2015) 195:4753–4759. doi: 10.4049/jimmunol.1501365

5. Dejbakhsh-Jones S, Okazaki H, Strober S. Similar rates of production of T and B lymphocytes in the bone marrow. *J Exp Med* (1995) 181:2201–2211. doi: 10.1084/jem.181.6.2201

6. Gatto D, Martin SW, Bessa J, Pellicioli E, Saudan P, Hinton HJ, Bachmann MF. Regulation of memory antibody levels: the role of persisting antigen versus plasma cell life span. *J Immunol Baltim Md 1950* (2007) 178:67–76. doi: 10.4049/jimmunol.178.1.67

7. Jones MA, DeWolf S, Vacharathit V, Yim M, Spencer S, Bamezai AK. Investigating B Cell Development, Natural and Primary Antibody Responses in Ly-6A/Sca-1 Deficient Mice. *PloS One* (2016) 11:e0157271. doi: 10.1371/journal.pone.0157271

8. Loder BF, Mutschler B, Ray RJ, Paige CJ, Sideras P, Torres R, Lamers MC, Carsetti R. B Cell Development in the Spleen Takes Place in Discrete Steps and Is Determined by the Quality of B Cell Receptor–Derived Signals. *J Exp Med* (1999) 190:75–90. doi: 10.1084/jem.190.1.75

9. Mackay F, Woodcock SA, Lawton P, Ambrose C, Baetscher M, Schneider P, Tschopp J, Browning JL. Mice transgenic for BAFF develop lymphocytic disorders along with autoimmune manifestations. *J Exp Med* (1999) 190:1697–1710. doi: 10.1084/jem.190.11.1697

10. Maqueda-Alfaro RA, Marcial-Juárez E, Calderón-Amador J, García-Cordero J, Orozco-Uribe M, Hernández-Cázares F, Medina-Pérez U, Sánchez-Torres LE, Flores-Langarica A, Cedillo-Barrón L, et al. Robust Plasma Cell Response to Skin-Inoculated Dengue Virus in Mice. *J Immunol Res* (2021) 2021:5511841. doi: 10.1155/2021/5511841

11. Moratz C, Hayman JR, Gu H, Kehrl JH. Abnormal B-cell responses to chemokines, disturbed plasma cell localization, and distorted immune tissue architecture in Rgs1-/- mice. *Mol Cell Biol* (2004) 24:5767–5775. doi: 10.1128/MCB.24.13.5767-5775.2004

12. Moreno RL, Sampson JS, Romero-Steiner S, Wong B, Johnson SE, Ades E, Carlone GM. A murine model for the study of immune memory in response to pneumococcal conjugate vaccination. *Vaccine* (2004) 22:3069–3079. doi: 10.1016/j.vaccine.2004.02.018

13. Ndungu FM, Cadman ET, Coulcher J, Nduati E, Couper E, Macdonald DW, Ng D, Langhorne J. Functional memory B cells and long-lived plasma cells are generated after a single Plasmodium chabaudi infection in mice. *PLoS Pathog* (2009) 5:e1000690. doi: 10.1371/journal.ppat.1000690

14. Pellegrini A, Guiñazú N, Aoki MP, Calero IC, Carrera-Silva EA, Girones N, Fresno M, Gea S. Spleen B cells from BALB/c are more prone to activation than spleen B cells from C57BL/6 mice during a secondary immune response to cruzipain. *Int Immunol* (2007) 19:1395–1402. doi: 10.1093/intimm/dxm107

15. Petkova SB, Yuan R, Tsaih S-W, Schott W, Roopenian DC, Paigen B. Genetic influence on immune phenotype revealed strain-specific variations in peripheral blood lineages. *Physiol Genomics* (2008) 34:304–314. doi: 10.1152/physiolgenomics.00185.2007

16. Robinson MJ, Dowling MR, Pitt C, O'Donnell K, Webster RH, Hill DL, Ding Z, Dvorscek AR, Brodie EJ, Hodgkin PD, et al. Long-lived plasma cells accumulate in the bone marrow at a constant rate from early in an immune response. *Sci Immunol* (2022) 7:eabm8389. doi: 10.1126/sciimmunol.abm8389

17. Schrock DC, Leddon SA, Hughson A, Miller J, Lacy-Hulbert A, Fowell DJ. Pivotal role for αV integrins in sustained Tfh support of the germinal center response for long-lived plasma cell generation. *Proc Natl Acad Sci U S A* (2019) 116:4462–4470. doi: 10.1073/pnas.1809329116

18. Slifka MK, Antia R, Whitmire JK, Ahmed R. Humoral immunity due to long-lived plasma cells. *Immunity* (1998) 8:363–372. doi: 10.1016/s1074-7613(00)80541-5

19. Slifka MK, Ahmed R. Long-lived plasma cells: a mechanism for maintaining persistent antibody production. *Curr Opin Immunol* (1998) 10:252–258. doi: 10.1016/s0952-7915(98)80162-3

20. Verheijen M, Rane S, Pearson C, Yates AJ, Seddon B. Fate Mapping Quantifies the Dynamics of B Cell Development and Activation throughout Life. *Cell Rep* (2020) 33:108376. doi: 10.1016/j.celrep.2020.108376

21. Vieira P, Rajewsky K. The half-lives of serum immunoglobulins in adult mice. *Eur J Immunol* (1988) 18:313–316. doi: 10.1002/eji.1830180221
